# Dental Ontogeny in Pliocene and Early Pleistocene Hominins

**DOI:** 10.1371/journal.pone.0118118

**Published:** 2015-02-18

**Authors:** Tanya M. Smith, Paul Tafforeau, Adeline Le Cabec, Anne Bonnin, Alexandra Houssaye, Joane Pouech, Jacopo Moggi-Cecchi, Fredrick Manthi, Carol Ward, Masrour Makaremi, Colin G. Menter

**Affiliations:** 1 Department of Human Evolutionary Biology, Harvard University, Cambridge, Massachusetts, United States of America; 2 ESRF—The European Synchrotron, Grenoble, France; 3 Department of Human Evolution, Max Planck Institute for Evolutionary Anthropology, Leipzig, Germany; 4 Paul Scherrer Institut, Swiss Light Source, Villigen, Switzerland; 5 Département Ecologie et Gestion de la Biodiversité, UMR 7179 CNRS, Muséum National d’Histoire Naturelle, Paris, France; 6 Laboratoire de Géologie, UMR 5276 CNRS, Université Claude Bernard Lyon 1, Villeurbanne, France; 7 Dipartimento di Biologia, Università di Firenze, Firenze, Italy; 8 Department of Earth Sciences, National Museums of Kenya, Nairobi, Kenya; 9 Department of Pathology and Anatomical Sciences, University of Missouri, Columbia, Missouri, United States of America; 10 Department of Orthodontics, University of Bordeaux II, Bordeaux, France; 11 Centre for Anthropological Research, University of Johannesburg, Johannesburg, South Africa; Monash University, AUSTRALIA

## Abstract

Until recently, our understanding of the evolution of human growth and development derived from studies of fossil juveniles that employed extant populations for both age determination and comparison. This circular approach has led to considerable debate about the human-like and ape-like affinities of fossil hominins. Teeth are invaluable for understanding maturation as age at death can be directly assessed from dental microstructure, and dental development has been shown to correlate with life history across primates broadly. We employ non-destructive synchrotron imaging to characterize incremental development, molar emergence, and age at death in more than 20 *Australopithecus anamensis*, *Australopithecus africanus*, *Paranthropus robustus* and South African early *Homo* juveniles. Long-period line periodicities range from at least 6–12 days (possibly 5–13 days), and do not support the hypothesis that australopiths have lower mean values than extant or fossil *Homo*. Crown formation times of australopith and early *Homo* postcanine teeth fall below or at the low end of extant human values; *Paranthropus robustus* dentitions have the shortest formation times. Pliocene and early Pleistocene hominins show remarkable variation, and previous reports of age at death that employ a narrow range of estimated long-period line periodicities, cuspal enamel thicknesses, or initiation ages are likely to be in error. New chronological ages for SK 62 and StW 151 are several months younger than previous histological estimates, while Sts 24 is more than one year older. Extant human standards overestimate age at death in hominins predating *Homo sapiens*, and should not be applied to other fossil taxa. We urge caution when inferring life history as aspects of dental development in Pliocene and early Pleistocene fossils are distinct from modern humans and African apes, and recent work has challenged the predictive power of primate-wide associations between hominoid first molar emergence and certain life history variables.

## Introduction

Evolutionary biologists have debated the evolution of human ontogeny for nearly a century [1–7], which continues to be fueled by discoveries of important new australopith fossils such as the Dikika baby [8] and the Malapa juvenile [9]. Contemporary discussions of the evolution of human life history largely stem from studies of dental development that began during the 1980s [1, 3, 4, 6]. Tooth microstructure is an important tool for the study of growth and development as a juvenile’s age at death can be precisely determined from fossilized dentitions without reliance on extant reference taxa, and histological age assessments may be accurate to within a few days or weeks [7, 10–13]. Christopher Dean and colleagues’ initial studies of incremental tooth growth in Plio-Pleistocene juveniles suggested that the duration of early hominin dental development was more ape-like than human-like [14, 15], which was interpreted as evidence that these hominins had an abbreviated period of dental growth and a shorter childhood than extant humans [16–18]. In 1989, Holly Smith demonstrated that molar emergence ages were correlated with aspects of life history, or the overall pace of growth and reproduction, in living primates [19]. First molar emergence ages in a small number of early hominins were estimated to be similar to living great apes [14, 15, 20], lending further support to the idea that early hominin life histories were more rapid than living humans. Since then, numerous scholars have cited these studies to suggest that early hominin life history was great ape-like [3, 6, 21–27], although as Dean and Lucas [24] note it is apparent that the great apes show considerable variation in their life histories [28]. Other scholars emphasize a more mosaic-like or unique pattern of life history in australopiths [2, 4], while some suggest the possibility of a more rapid life history in early hominins than in the great apes [16, 29].

Exploring the evolution of life history is of particular significance for understanding unique attributes of extant humans, such as early weaning ages, long childhoods, and extended post-reproductive periods. Furthermore, the fossil record provides scant evidence to assess the rate and duration of neurological or skeletal developmental beyond the information recorded in teeth. There are no alternative methods available to age juveniles without employing extant populations, which leads to circular reasoning when comparing fossil to modern taxa. This problem of circularity is particularly apparent in the history of debates about the *Australopithecus africanus* Taung child, which was often aged at ~3 or ~6 years according to ape and human developmental standards, respectively [4, 24, 30–33]. In the following study we present precise estimates of dental development and age at death in Pliocene and Pleistocene hominins that do not rely on parameters estimated from living taxa. We aim to establish the nature of similarities and differences in dental development among early hominins, extant humans, and African apes by applying non-destructive synchrotron imaging to a diverse sample of East and South African fossil juveniles.

### Tooth Growth in Early Hominins

Enamel and dentine formation are characterized by the production of long- and short-period increments that represent the position of the forming front and the daily products of the secretory cells, respectively. Long-period features in enamel are known as Retzius lines, and are equivalent to Andresen’s lines in dentine. External manifestations of long-period lines on tooth crowns and roots are known as perikymata (enamel) and periradicular bands (dentine), respectively [34–36]. Short-period lines are known as cross-striations and laminations, equivalent to von Ebner’s lines in dentine [37–40]. Cross-striations, laminations, and von Ebner’s lines show a 24-hour frequency [39, 41–43]. Cross-striations are used as a standard to determine the periodicity of long-period lines in both tissues because they are easier to image than von Ebner’s lines [37]. The periodicity of enamel and dentine long-period lines is consistent within a single tooth and in all teeth belonging to the same individual, although this may vary within a taxon [44]. Importantly, counts and measurements of long- and short-period lines provide information on the rate and duration of enamel and dentine secretion, which may be combined to determine the crown formation time, rate and duration of root extension, and age at death in developing dentitions.

Bromage and Dean’s innovative study [14] of incremental dental development in Pliocene and early Pleistocene hominins was an important advance in our understanding of the evolution of human development. They reported ages at death for six juveniles from counts of external long-period lines (perikymata) and estimates of internal growth parameters (long-period line periodicity and cuspal enamel formation time). Their study yielded ages that were markedly younger than those predicted from extant human growth standards, but similar to those of great apes. For *A*. *africanus*, their age at death for Sts 24 was subsequently used to infer that Taung was also approximately 3.3 years of age, as it died at a similar developmental stage, although this has subsequently been revised to 3.7–3.9 years based on information from StW 402 [45]. Assessments of Pliocene and early Pleistocene hominin enamel formation have continued, and as was the case for Bromage and Dean [14], these studies have generally been limited to quantification of external features or those in naturally-fractured teeth due to the destructive nature of more comprehensive traditional histological analyses [46, 47]. Several of these early reports of crown formation times derived from naturally fractured teeth [48–50] tended not to specify the tooth and or cusp type, impairing comparisons as crown formation times vary among the cusps within a molar, as well as within a cusp type across serial molars [51].

Although daily secretion rates (cross-striation spacing), enamel long-period line (Retzius line or perikyma) numbers, and long-period line periodicities have been documented in numerous Pliocene and early Pleistocene hominin teeth [52, 53], there are few secure data on crown formation times, including initiation and completion ages, limiting estimates of age at death and molar emergence. Given the historic necessity of estimating missing data when histological sectioning is not possible [14, 21, 45, 52], fossil hominin ages at death have considerable potential to be inaccurate [29, 54] Here we employ synchrotron virtual imaging [55] to comprehensively characterize dental development (long-period line periodicity, cuspal enamel thickness, crown formation time, initiation age, tooth calcification stage) and age at death in a diverse sample of more than 20 Pliocene and early Pleistocene hominin juveniles. Counts of long- and short-period incremental lines are combined to determine crown formation time, molar emergence age, and age at death in developing dentitions. A substantial advantage of using non-destructive virtual histology is that internal “section planes” can be precisely controlled [54, 55], improving the accuracy of counts and measurements of incremental features, as well as formation time and age at death determination. Assessments of age at death from tooth microstructure based entirely on species-specific developmental variables are now possible, allowing scholars to avoid the circular logic of comparing estimates of fossil hominin dental development that employ extant great ape and human developmental variables to these same reference species.

## Materials and Methods

### Sample and Data Acquisition

**Fig 1:**
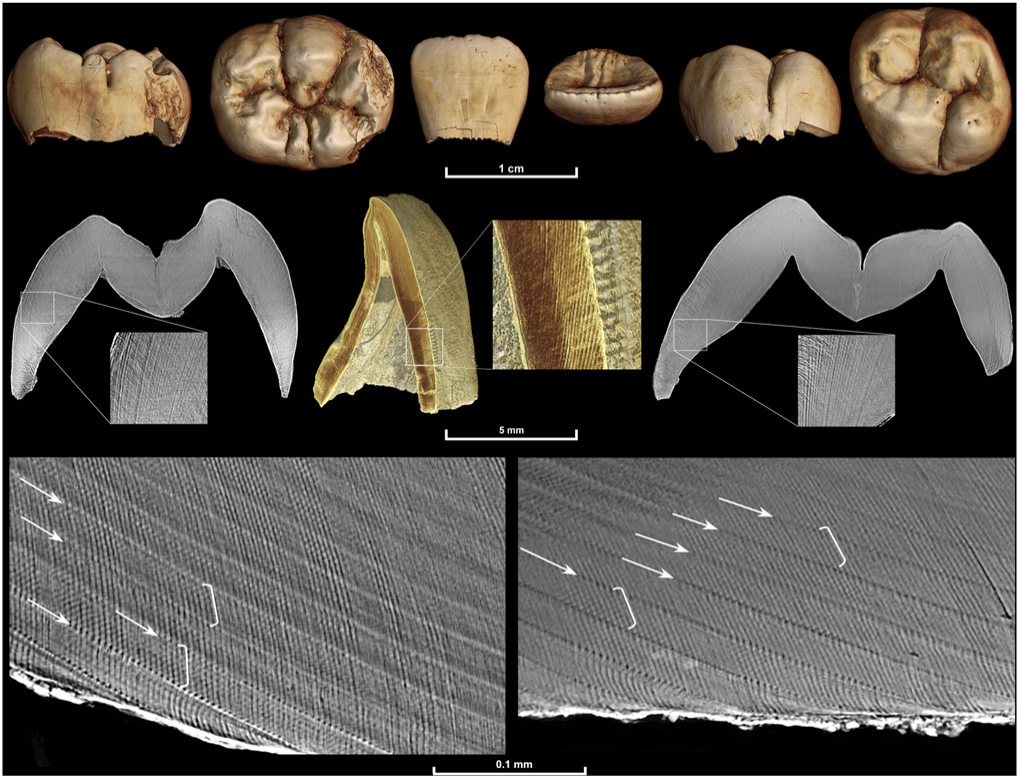
Multiscale synchrotron imaging of a fossil *Homo* juvenile individual. DNH 67 (right lower first molar: left), DNH 71 (right upper central incisor: middle), and DNH 70 (left upper first molar: right). Images are from scans performed with the following voxel sizes: 20 μm (upper row), 5 μm (middle row), and 0.7 μm (lower row; DNH 67: left, DNH 70: right). An identical internal developmental defect pattern confirmed that the two molars, which were found isolated but in close proximity, came from the same individual. They also both show an identical long-period line periodicity of 8 days, as 8 light and dark bands (cross-striations illustrated in white brackets) can be seen between successive long-period lines (Retzius lines illustrated by white arrows). It was not possible to determine the age death for this individual due to postmortem loss of the incisor cervix and dentine from all teeth.

The fossil sample includes juvenile individuals attributed to *Australopithecus anamensis*, *A*. *africanus*, *Paranthropus robustus*, South African early *Homo*, and two individuals of uncertain taxonomic attribution (StW 151; ref. [56]; KB 5223: ref. [57]) (Table 1). Specimens are derived from the Kenyan site of Kanapoi, and the South African sites of Sterkfontein, Makapansgat, Swartkrans, Kromdraai, and Drimolen, and morphological descriptions and/or illustrations of these specimens have previously been published [56–66]. Isolated teeth and intact jaws were scanned using propagation phase contrast X-ray synchrotron microtomography on beamline ID19 of the European Synchrotron Radiation Facility (Grenoble, France) following acquisition parameters detailed in ref. [67]. We used a non-destructive multiscale approach (voxel sizes ranging from 30 to 0.7 μm) to assess overall tooth formation and fine incremental features with VGStudioMax 2.1/2.2 software (Volume Graphics, Heidelberg, Germany) (Fig. 1).

Long-period incremental lines, neonatal (birth) lines, and developmental defect patterns were imaged on complete teeth with voxel sizes around 5 μm [54, 55]. In certain instances when internal long-period lines were difficult to count due to poor preservation, specimens were assessed by aligning 2D slices with 3D renderings of the tooth surface and enamel-dentine junction, which were produced with a combination of colored grazing incidence light sources following ref. [67]. This method involves capturing the phase contrast fringes at the outer enamel surface and enamel-dentine junction through segmentation of the structural interfaces, and generation of three-dimensional models with Phong’s algorithm and virtual lighting, which enhances surface topography and variation in tissue density. The 3D renderings and virtual slices employed in this study are available in the open access database for paleontology hosted by the ESRF (http://paleo.esrf.eu).

**Table 1:** Hominin material included in this study.

Taxon	Accession	Material Scanned at 5 microns	Available Dental Sample
*Australopithecus anamensis*	KNM-KP 31712	RLC, LLM1, RLM1	mandibular fragments and associated teeth
	KNM-KP 34725	RUM2, LLI1, RLI2, RLC, RLM1, LLM2	associated maxillary and mandular teeth
*Australopithecus africanus*	Sts 2	LUC, LUdP4, LUM1	maxilla and associated teeth
	Sts 24	RUI1, LUI2, RUC, LUP3, RUP3, RUM1, RLI1, LLI2, RLM1	maxilla and associated mandibular teeth
	MLD 2	LLC, LLP3, LLP4, LLM1, LLM2	mandible
	MLD 11/30	RUI2, RUC, RUP3, RUP4, RUM1	maxilla and associated teeth
*Paranthropus robustus*	SK 61	RLI1, RLI2, LLI2, LLC, LLP3, RLM1	mandible
	SK 62	LLI1, LLI2, LLC, LLM1, LLM2	mandible
	EM 2368	(only scanned at 0.7 microns)	molar crown fragment from Swartkrans
	TM 1536	RLI1, RLI2, RLM1	mandible
	DNH 44	RLI1, RLM1	mandible
	DNH 47	LUdP4, RUI1, LUM1	maxilla and associated teeth
	DNH 60	LUM1, RLM1, RLM2	associated teeth with skull
	DNH 84	RUI1, LUI1, LUM1	maxilla and associated teeth
	DNH 101	RLM2	isolated molar
	DNH 107	RLI1, LLC, RLM1	associated teeth
	DNH 108	LUC, RUC, LUP3, RUP3, LUM1, RUM1, RUM2	associated teeth
*South African Homo*	DNH 35	RLM1	mandible
	DNH 39	RUM1	isolated molar
	DNH 62	LUM1	isolated molar
	DNH 67/70/71	RLM1/LUM1/RUI1	associated teeth
	DNH 83	RUdP4	maxilla
	DNH 100	LLM2	isolated molar
Indeterminate hominins	StW 151	LUC, LUM1, RUM2, LLC, LLM1	partial max and mand and associated teeth
	KB 5223	LII1, LLI2, RLI1, RLI2, LLM1	associated teeth

Tooth types: R—right, L—left, U—upper, L—lower, I—incisor, C—canine, P—premolar, M—molar, dP—deciduous premolar. Many of these teeth had yet to complete crown formation prior to death, and none of the fossils in this study possessed associated third molars.

In order to characterize long-period line periodicity, selected areas of one or more teeth of each individual were scanned using sub-micron resolution local phase contrast X-ray synchrotron microtomography, and virtual histological slices (typically 30 to 100 μm thick) were produced following 3D optimized orientation (Fig. 1). Temporary darkening of the enamel occurred in some areas scanned at high resolution; teeth were returned to their original color with 1–12 hours of low energy UV illumination (70 W dark light neon tube, 370 nm wavelength; following ref. [55].)

### Developmental Feature Quantification

Cuspal enamel thickness was measured on 2D slices with Adobe Photoshop CS5 from the dentine horn tip to the approximate position of the first-formed long-period line (perikyma) at the crown surface. Labio-lingual sections were cut for anterior teeth, buccal-lingual sections were cut for premolars, and buccal-lingual sections were cut for the mesial and distal cusps of molars following a standardized virtual orientation technique [54]. Long-period line periodicity was determined through counts of daily growth lines between successive long-period lines in enamel (Fig. 1). The Mann-Whitney *U* test was employed for comparisons of periodicity values between taxa represented by four or more individuals. The fidelity of virtual histology has been demonstrated through comparisons of virtual and histological sections [55], and long-period line periodicity values determined using this approach have been shown to be equivalent to those determined using light microscopy [68].

Crown formation time was calculated as the sum of cuspal and lateral formation times. Cuspal formation time was typically calculated by dividing the linear enamel thickness of each tooth cusp by the species-specific average cuspal daily secretion rates in ref. [53]. Daily secretion rates for KB 5223 were taken from ref. [57]. Direct measurements of daily secretion rates in DNH 35 were made in the inner, middle, and outer cuspal enamel of the mesiolingual cusp from 0.7 μm scans. Direct measurements of cuspal daily secretion rates for other specimens were not possible due to the time-intensive nature of quantifying cross-striation spacing in cuspal enamel. The average value from DNH 35 (6.06 μm/day) was used to estimate cuspal enamel formation times in other South African early *Homo* samples. For Stw 151, which has been attributed to either early *Homo* or *A*. *africanus* [56], estimates of cuspal enamel formation time were made using secretion rates from DNH 35 and average values from *A*. *africanus* [53], resulting in differences of 18–50 days depending on the tooth analyzed. Lateral enamel formation time was calculated by multiplying the number of long-period lines (Retzius lines and/or perikymata) by the long-period line periodicity. Minor estimates of long-period lines were made for worn or chipped enamel; specimens were excluded when long-period lines could not be counted on at least 90% of the total crown height. Comparative data on long-period line periodicity and crown formation times were chosen to minimize potential bias from methodological differences or interobserver error, and data sources are provided in the corresponding tables.

### Age at Death, Initiation, and Calcification Stage Determination

Age at death was typically calculated by identification of the neonatal (birth) line in permanent first molars (M1s) or deciduous premolars, and summation of subsequent crown and root formation determined from incremental features. In three individuals (DNH 35, DNH 84, and KB 5223) it was not possible to identify a neonatal line, and thus the M1 cusp employed was assumed to have initiated at birth, as species-specific initiation ages were not available for these particular cusps. For older juveniles, developmental defects (hypoplasias or accentuated lines) were registered across the dentition to link developmentally-overlapping teeth [10, 36, 54]. In four individuals (MLD 11/30, SK 62, TM 1536, and DNH 44) it was not possible to register M1s with subsequent teeth. In these cases initiation ages of anterior teeth from individuals of the same species were used to calculate age at death by adding crown and root formation times to the tooth-specific initiation ages. Initiation ages were determined by registering synchronously forming teeth with developmental defects and subtracting preceding developmental time from the age of the defect used to register teeth. For example, an accentuated line was identified at 143 days of age in the mandibular M1 of KNM-KP 31712, which was also observed in the mandibular canine 110 days after initiation and 878 days before death. Thus the mandibular canine initiated at 33 days of age (143 days minus 110 days) and the individual died at 1021 days of age (33 days of age at initiation plus 988 days of canine crown formation until death).

Tooth calcification was determined from virtual 2D sections of each tooth for those individuals whose age could be determined. The section planes employed to assess calcification stages often differed from those employed to assess cuspal enamel thickness. Sections were cut to optimize developing root profiles for calcification stage determination, and a numerical score was assigned to each tooth following the commonly employed classification system of Moorrees and colleagues [69]. Calcification standards derived from this 14 stage system [69] are more precise than those derived from Demirjian and colleagues’ 8 stage system [70], as crown and root formation is represented by more discrete developmental stages in the former case. Ages were assigned to each individual tooth using an average of the extant human male and female standards in ref. [71]. We employed Anderson and colleagues’ calcification tables [71] as they include maxillary and mandibular standards for each tooth of human children from ages 3 years and up. We also collected comparative calcification data from panoramic X-rays of European and North African human children (an expanded sample originally detailed in ref. [54]), and from recently deceased known-age West African wild chimpanzees [72].

While additional aging systems from extant human tooth calcification and/or emergence studies are available [73, 74], the tables in ref. [71] allow discrete age calculations for hominins represented by partial dentitions and for whom emergence status may be unknown. Although scholars have noted limitations in the accuracy of this and other aging methods [73, 75], as well as potential population-level variation in extant human tooth calcification ([76] but see refs. [77, 78]), the aim of our study was to apply a consistent method to extant humans, chimpanzees, and juvenile hominins represented by partial dentitions in order compare the ontogeny of tooth calcification among these groups. Any bias in the accuracy of the calcification system or aging standards we employ here applies equally to each group and does not impact conclusions based on relative patterns. Finally, in order to assess the appropriateness of chimpanzee calcification stages for hominin age prediction, we applied Kuykendall’s [79] predictive standard (requiring the presence of all mandibular teeth except M3) to the two *P*. *robustus* juveniles that possessed the appropriate teeth and for which it was possible to estimate age at death histologically.

## Results and Discussion

### Long-period Line Periodicity, Enamel Thickness, and Crown Formation Time

**Fig 2:**
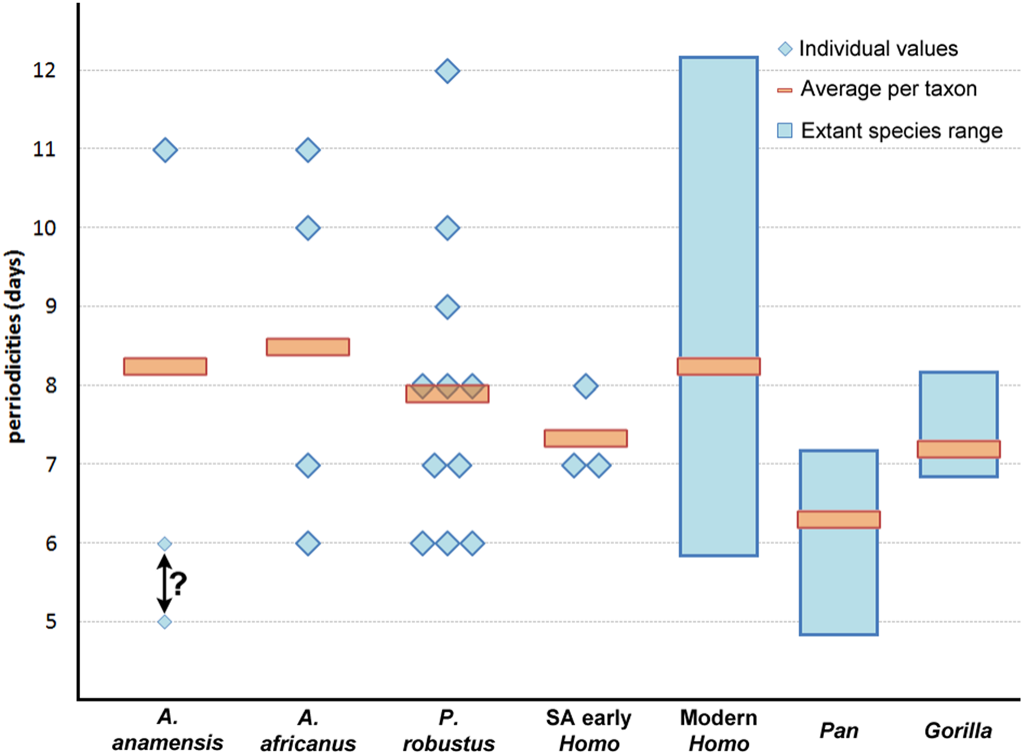
Long-period line periodicity values in Pliocene and early Pleistocene hominins, extant humans, and African apes. Two values (5 or 6 days) are presented for *A*. *anamensis* KNM-KP 34725 due to some uncertainty (indicated by “?”), as shown in Fig. B in S1 File. Individual hominin values and sample sizes of extant taxa are given in Tables A and B in S1 File, respectively.

We find a remarkably wide range of long-period line periodicity values in 22 Pliocene and early Pleistocene hominins, which range from at least 6–12 days, and possibly 5–13 days (Fig. 2, Table A in S1 File). In two individuals it was not possible to choose between two periodicity values (KNM-KP 34725: 5 or 6 days, KB 5223: 12 or 13 days); subsequent calculations of crown formation time and age at death for these individuals are given for each periodicity value. Periodicity values in the 11 *Paranthropus robustus* individuals range from 6–12 days, and do not show a significant difference when compared with *Australopithecus africanus*, *Homo neanderthalensis*, fossil *Homo sapiens*, extant humans, gorillas, or previously reported values for this species (Tables B-C in S1 File). Both *P*. *robustus* and *A*. *africanus* periodicity values are significantly greater than chimpanzees. Australopiths (including *P*. *robustus*) do not show smaller ranges or lower average periodicity values than extant or fossil *Homo* (*contra* [53]). Previous studies of australopiths have reported periodicities of 6–9 days (n = 29 individuals) (Table B in S1 File). When considered in totality, australopith periodicity values of 6–9 days are more common than values of 10–12 days, as is the case with extant humans [51].

Cuspal enamel thickness values vary markedly among Pliocene and early Pleistocene hominins, ranging from thin enamel in *Australopithecus anamensis* to extremely thick enamel in *P*. *robustus* (Fig. A and Table D in S1 File). These differences lead to variation in cuspal formation times, although less so than would be expected as the taxa with the thickest and thinnest enamel appear to show the fastest and slowest daily secretion rates, respectively [53]. For example, mandibular M1 and second molar cuspal enamel thickness values are roughly twice as great in *P*. *robustus* than in *A*. *anamensis*, yet average cuspal daily secretion rates are reported to be ~20% greater in *P*. *robustus* than in *A*. *anamensis* [53]. Because the cuspal enamel formation time is estimated from division of the linear enamel thickness by the average daily secretion rate, these rate differences result in formation times that differ by less than a factor of two in this comparison. In general, our cuspal enamel thickness values are broadly similar to earlier reports of *A*. *anamensis*, *P*. *robustus*, *A*. *africanus*, and early *Homo* molars [65, 80–82], although direct comparisons are limited due to methodological differences.

**Fig 3:**
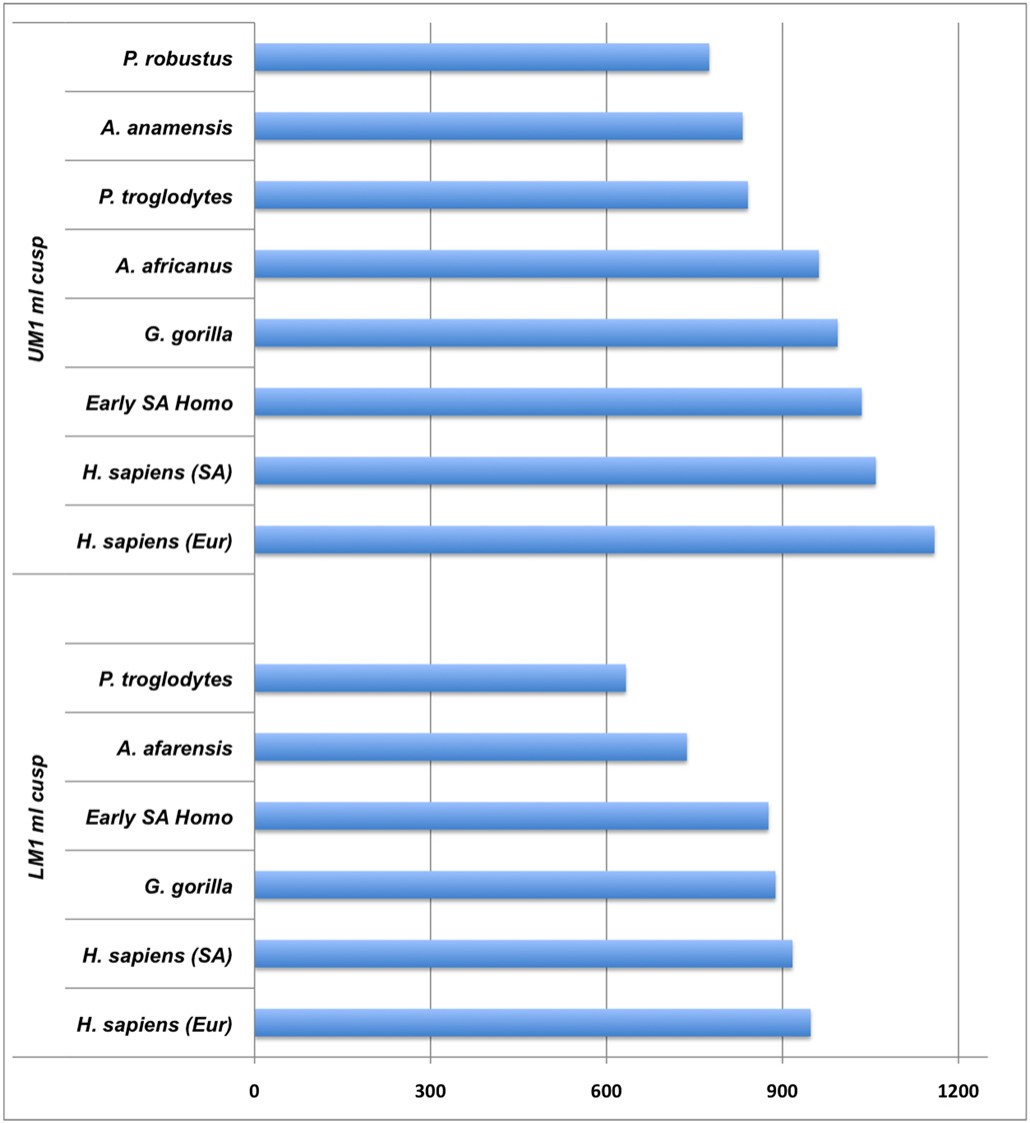
Average molar crown formation times (in days) in early hominins and extant apes and humans. UM1 ml cusp = maxillary first molar mesiolingual cusp; LM1 ml cusp = mandibular first molar mesiolingual cusp. UM1 ml cusp of *A*. *anamensis* from ref. [65]; LM1 ml cusp of *A*. *afarensis* from ref. [47]. Extant comparative data sources are given in Table 2.

Hominin incisor formation times are shorter than chimpanzees (*contra* [6]), and are more similar to those of extant humans and gorillas (Table 2). This is likely due, in part, to absolute differences in tooth size among these taxa, which are most extreme in the diminutive anterior dentition of *P*. *robustus*. Maxillary third premolar (P3) formation times in *A*. *africanus* and *P*. *robustus* are shorter than those of extant humans and chimpanzees, while *A*. *africanus* mandibular premolar values exceed mean values for extant humans from South Africa (P3 buccal cusp) and chimpanzees (P4 lingual cusp). Comparisons of molar crown formation times also show a complex pattern, which is difficult to generalize as each molar tooth and cusp must be considered independently [51]. Our new australopith molar crown formation times are broadly consistent with previous reports of *A*. *anamensis*, *Australopithecus afarensis*, *A*. *africanus*, *P*. *robustus*, and *Homo erectus* values [15, 21, 47, 65, 82], which are shorter than those of extant humans [54, 83]. In contrast, molar crown formation times for the South African early *Homo* individual DNH 67/70/71 are most similar to times for both gorillas and extant humans from South Africa (Fig. 3), underscoring the importance of further recovery and histological study of this enigmatic hominin group. Given the small sample sizes of the available fossil and comparative material, it is premature to draw firm conclusions about differences in crown formation times among these taxa. Molar crown formation times, for example, are known to vary by ~ 6–12 months in small samples of African apes and extant humans [10, 54, 84], and comparable standard deviations have also been reported for great ape and human canine formation times [85].

**Table 2:** Average crown formation times (in days) determined by histological methods.

Tooth /cusp	*H*. *sapiens* (Eur)	*H*. *sapiens* (SA)	Pan troglodytes	Gorilla gorilla	A. anamensis	A. africanus	P. robustus	SA *Homo*	Stw 151	KB 5223
UI1	1582	1318	1827	1261 (2)	—	1557	—	—	—	—
UI2	1427	1324	1461	1496	—	961	—	—	—	—
UC	1613 (mixed sex)	1438 (mixed sex)	2053 (sex unknown)	—	—	1014	818	—	>1562/1580	—
UP3 buc	1407	1113	1401	—	—	—	770	—	—	—
UP3 lin	1281	972	967	—	—	826	—	—	—	—
UM1 mb	1017	981	867 (6)	784	—	544	612	—	—	—
UM1 ml	1159	1059	775 (5)	994 (2)	—	962	775	1035	—	—
UM1 dl	1088	—	684 (2)	1168 (2)	—	959	785	—	952/986	—
UM2 ml	1141	1218	1012 (2)	1057	—	—	806	—	—	—
UM2 dl	1068	—	—	1136	899/1001	—	—	—	921/971	—
										
LI1	1251	1096	1581	1141 (2)	1170/1381	1220	891 (2)	—	—	—
LI2	1293	1169	1764	1287 (2)	—	1399	—	—	—	1296/1389
LC	2004 (mixed sex)	1721 (sex unknown)	2135 (f:6), 2486 (m:6)	2058 (f:7), 3117 (m:8)	—	—	>1558	—	—	—
LP3 buc	1454	1157	1496	—	—	1223	—	—	—	—
LP4 lin	—	—	823 (2)	—	—	1056	—	—	—	—
LM1 mb	1097	1096	797 (6)	932 (2)	—	—	949	—	—	—
LM1 ml	948	917	633 (5)	905 (3)	—	—	—	876	—	—
LM1 db	1111	—	811 (8)	1142 (2)	—	1052	—	—	—	—
LM1 dl	939	—	662 (4)	860 (2)	—	813 (2)	832	675	—	1110/1164
LM2 mb	1096	1145	1009 (5)	1241	—	1013	911	—	—	—
LM2 ml	904	931	847 (6)	964 (2)	754/830	996	—	—	—	—
LM2 db	—	—	1160	1118 (3)	—	—	960	—	—	—

Tooth/cusp types: U—upper, L—lower, I—incisor, C—canine, P—premolar, M—molar, buc—buccal cusp, ling—lingual cusp, mb—mesiobuccal cusp, ml—mesiolingual cusp, db—distobuccal cusp, dl—distolingual cusp. *Homo sapiens* European (EUR) and South African (SA) ranges and sample sizes given in refs. 54, 83. *Pan troglodytes* data modified from refs. [72, 84, 85, 88, 104]; *Gorilla gorilla* data from refs. [10, 89, 105, 106]; samples sizes for extant apes are given in parentheses when greater than one. Fossil hominin sample sizes are given in parentheses when greater than one; *A*. *anamensis* values reported for periodicity of 5 or 6 days in KNM-KP 34725; StW 151 values based on cuspal daily secretion rates of 5.53 um/day (*A*. *africanus*) from ref. [53] and 6.06 um/day (early *Homo*, determined from this study); KB 5223 values based on cuspal daily secretion rates from ref. [57] and long-period line periodicity values of 12 or 13.

### Age at Death and Molar Eruption

These data facilitate histologically-derived age at death estimates for 16 hominin juveniles (Table 3; Table E in S1 File). Previous histological studies reported ages at death for Sts 24 at 3.3 years [14], SK 62 at 3.35–3.48 years [14], and StW 151 at 5.2–5.3 years [66]; our new ages differ by 0.2–1.1 years depending on the individual. Age at death estimates in other early hominins [14, 17, 20, 24, 29, 32, 45] should be reconsidered in light of these findings, particularly given newly expanded ranges of long-period line periodicity values in australopiths. For example, the *A*. *afarensis* specimen LH 2 was estimated to have died at 3.25 years of age [14] based, in part, on a count of 130 long-period lines for the mandibular central incisor and an estimated periodicity value of 7 days. If we assume that *A*. *afarensis* possessed a similar range of long-period line values to *A*. *anamensis* and *A*. *africanus* (minimally 6–11 days; Fig. 2), age at death estimates would range from 130 days younger (6 day periodicity) to 520 days older (11 day periodicity) than the original estimate. These potential age ranges complicate comparisons with extant taxa and underscore the necessity of comprehensive physical or virtual histological analyses for precise age at death estimation.

**Table 3:** Age at death for juvenile hominins based on incremental dental development.

Taxon	Specimen	Years
*Australopithecus anamensis*	KNM-KP 31712	2.80
	KNM-KP 34725	3.63 (5)/4.25 (6)
*Australopithecus africanus*	Sts 2	2.52
	Sts 24	4.35
	MLD 11/30	3.42
*Paranthropus robustus*	SK 62	3.12
	TM 1536	1.63–2.02
	DNH 44	1.70
	DNH 47	0.67–0.77
	DNH 84	2.24
	DNH 107	4.82
	DNH 108	5.35/5.53
South African *Homo*	DNH 35	2.18
	DNH 83	0.52 (7)/0.59 (8)
Indetermined	StW 151	4.62/4.70
	KB 5223	5.16–5.45 (12)/5.41–5.71 (13)

Details for individual calculations are given in Table E in S1 File. Ages for KNM-KP 34725, DNH 83, and KB 5223 are given for multiple periodicity values shown in parentheses (which should be considered alternative ages rather than error ranges). The age range for each periodicity value in KB 5223 also reflects some uncertainty in the age of an accentuated line matched across the dentition. The age range for TM 1536 represents estimates derived from lower central incisor initiation ages for two *P*. *robustus* specimens. The age range for DNH 47 reflects some uncertainty in postnatal long-period line number. The age range for DNH 108 reflects some uncertainty in the age of an accentuated line matched across the dentition. Due to taxonomic uncertainty, the age for StW 151 is based on estimates of average cuspal enamel secretion rates from *A*. *africanus* (from ref. [53]) and South African *Homo* (measured directly during this study), which should be considered alternative ages rather than error ranges.

**Fig 4:**
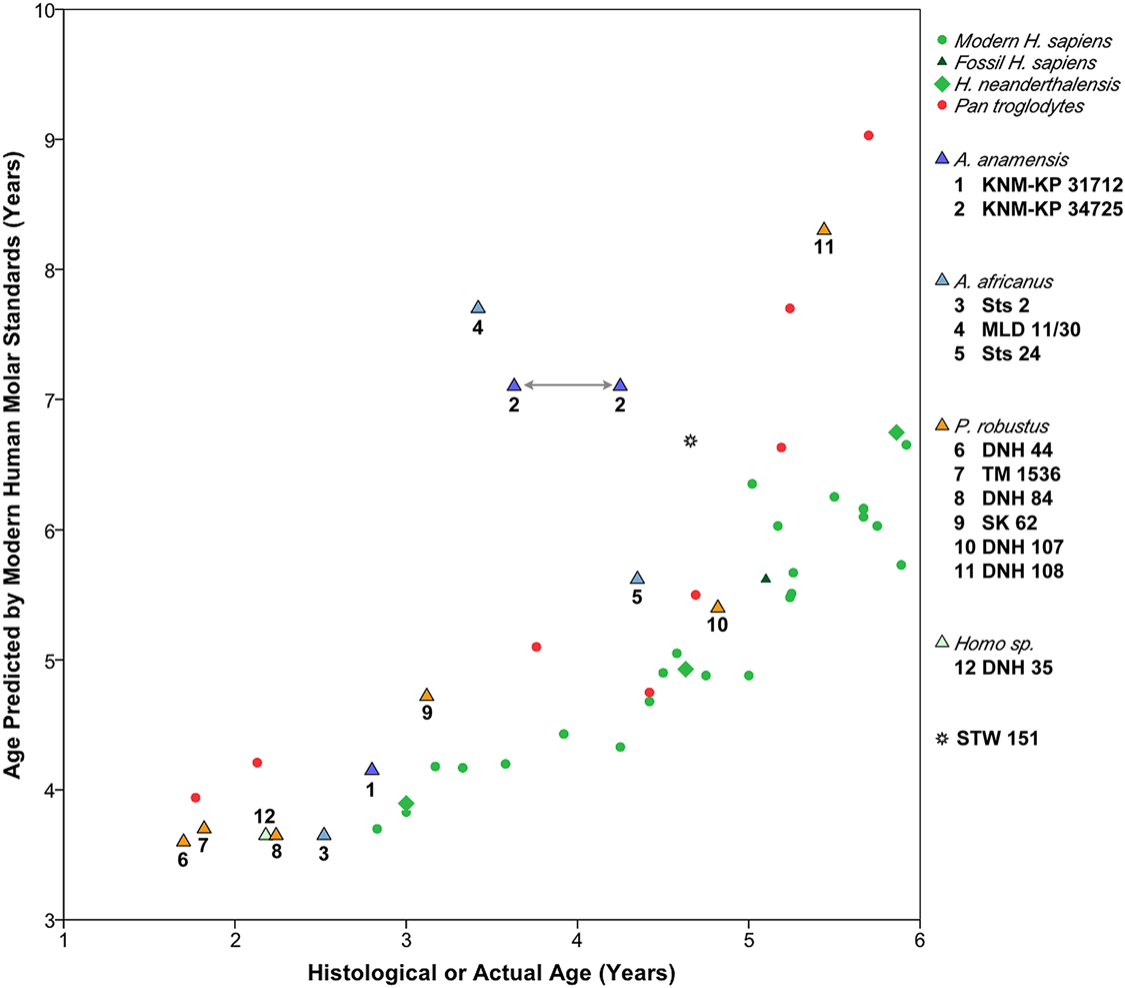
Ages predicted from extant human molar calcification standards compared to known- or histologically-derived ages. Two values are presented for *A*. *anamensis* KNM-KP 34725 due to uncertainty in the periodicity value. Data on extant human children derive from panoramic X-rays of known-age European and North African children, representing an expanded sample originally detailed in [54]. Fossil *Homo sapiens* and *Homo neanderthalensis* samples are from [54]; *Pan troglodytes* are known-age wild western chimpanzees [72].

Since our age at death estimates do not rely on extant human or great ape developmental information, we compared ages derived from the calcification stage of each tooth of each individual (Figs. C-R in S1 File) to a sample of known-age extant humans and wild chimpanzees (Fig. S in S1 File). Because chimpanzee anterior tooth and premolar morphology differs markedly from fossil hominins and extant humans, we also assessed the developmental status of individuals based solely on molar calcification (Fig. 4). Pliocene and early Pleistocene hominins followed an ontogenetic schedule of molar development that is more similar to chimpanzees than to extant humans, as has been inferred from assessments of other Pliocene and early Pleistocene hominins [14, 15, 20, 45]. However, marked differences are apparent for one *A*. *anamensis* (KNM-KP 34725) and one *A*. *africanus* individual (MLD 11/30), which show particularly rapid dental development that exceeds similarly-aged humans and chimpanzees. Age at death was also predicted for two *P*. *robustus* individuals with captive chimpanzee standards [79]. The resulting age estimates were closer to the actual histological ages than the ages predicted from extant human standards for only one of two cases (Table F in S1 File). In summary, radiographic developmental standards from extant humans and chimpanzees do not yield consistent or accurate ages at death estimates for Pliocene or early Pleistocene hominins.

None of the individuals in this study appear to have died during the initial skeletal phase of M1 emergence (alveolar eruption). Marked wear facets are apparent on the mandibular M1 of a 3.6 or 4.2 year-old *A*. *anamensis* individual (KNM-KP 34725) and a 3.4 year-old *A*. *africanus* individual (MLD 11/30). More subtle minor wear facets were also observed on the maxillary and mandibular M1s of a 4.4 year-old *A*. *africanus* individual (Sts 24), implying variation in *A*. *africanus* M1 emergence that is comparable to chimpanzees, which may erupt their first molars between ~2.1–4.5 years of age [72, 84, 86–88]. Three *P*. *robustus* individuals sampled here are also of interest for consideration of M1 emergence ages: 3.1 year-old SK 62 had not yet erupted its M1s (although the right mandibular I1 was erupting); the mandibular M1 of DNH 107 appeared to be nearing full occlusion (with slight facets) at 4.8 years of age (Fig. T in S1 File), and the maxillary M1 of DNH 108 showed marked facets by 5.4 or 5.5 years of age. Remarkably, the 4.6 or 4.7 year-old StW 151 individual (attributed to either early *Homo* or *A*. *africanus*; ref. [56]) appears to be more developmentally advanced than the 4.8 year-old *P*. *robustus* individual DNH 107, which showed less wear on its mandibular M1 (and had yet to erupt its right mandibular I1.) The maxillary M1 emergence status and wear in StW 151 appears to be more similar to the 5.4 or 5.5 year-old *P*. *robustus* individual DNH 108. Although it is difficult to precisely compare emergence among these individuals, our results suggest that M1 emergence in *A*. *anamensis* and/or *A*. *africanus* may have been somewhat earlier than in *P*. *robustus*.

## Conclusions

Substantial intraspecific variation exists in aspects of dental development quantified in this study (long-period line periodicity and cuspal enamel thickness), which has also been demonstrated for extant human populations and African apes [10, 51, 54, 72, 83, 84, 89, 90]. The range and frequencies of long-period line periodicity values in australopiths are comparable to a large sample of extant humans [51]. Bromage and colleagues [91] argue that long-period line periodicity represents the expression of a physiological oscillator that controls the pace of life history. Long-period line periodicity shows a positive correlation with body mass across primates and mammals more broadly [46, 92], and this relationship has been hypothesized to be driven by selection on body mass [91]. However expected relationships among life history variables, body mass, and periodicity may not hold among closely related primates (or among individuals within a species). For example, lowland gorillas have significantly greater periodicity values than common chimpanzees (Z = -4.273, p < 0.001), yet chimpanzees have longer interbirth intervals, and later ages at weaning, first reproduction, and first birth [93–96]. Similar concerns have been raised about the widely cited relationship between hominoid molar emergence and life history [5, 86, 97, 98], which is discussed further below.

Pliocene and early Pleistocene hominin incisor, canine, and premolar crown formation times reveal considerable interspecific variation that warrants further study, particularly with regard to potential sex differences and constraints due to absolute tooth sizes. New molar crown formation times are broadly comparable to previous reports of fossil hominins, and are typically shorter than molar crown formation times in extant humans. We have also documented relatively late initiation ages for certain *P*. *robustus* teeth (Table G in S1 File), which impact estimates of age at tooth crown formation and emergence. For example, mandibular incisor initiation in *P*. *robustus* occurred between 109–251 days, which is approximately one to five months later than the modern human value employed by Bromage and Dean [14]. Previous histological assessments of age at death (and molar emergence age) in early fossil hominins should be reconsidered in light of the expanded range of long-period line periodicities, cuspal enamel thickness values, crown formation times, and initiation ages documented here.

We present histologically-derived ages at death for 16 Pliocene and early Pleistocene hominins, illustrating an approach that may eventually lead to the development of australopith calcification standards in order to estimate the age of other juveniles. Importantly, these ages were determined from developmental information quantified directly from the specimens themselves and supplemented with species-specific information on daily secretion rates [53, 57] and initiation ages from this study when necessary. This facilitates discrete developmental comparisons with extant African apes and humans, and suggests a more nuanced interpretation of the developmental affinities of Pliocene and early Pleistocene hominins. Analyses of rare histological (physical) sections posited that australopith dental development (daily secretion rate, age at M1 emergence, root extension rate) was similar to living great apes and more rapid than extant humans, implying that early hominin life histories were great ape-like [6, 15, 21, 24]. Our comparisons with extant humans and African apes demonstrate a complex pattern of similarities and differences in long-period line periodicity, crown formation time, the timing of tooth calcification, and molar eruption ages, prohibiting simplistic characterizations of these species as either “human-like” or “great ape-like.” While some specialists of tooth development have reached similar conclusions over the past decade [2, 4], initial determinations of age at death for juvenile hominins continue to be made from extant ape developmental standards (e.g., *Australopithecus afarensis* Dikika child: ref. [8]). Moreover, this understanding is complicated by reports that dental development in later hominins (e.g., *Homo erectus* Narikotome KNM-WT 150000) may resemble that of African apes [21, 99], which underscores the need for comprehensive histological assessments based entirely on species-specific developmental information.

Studies of dental development have also been of great interest for reconstructing the evolution of human life history, although scholars have recently begun to question the association between molar emergence and life history variables among extant apes and humans [5, 7, 86, 97]. For example, fundamental questions remain about the suitability of primate-wide trends in M1 emergence for predicting weaning ages in hominoids. While it is likely that M1 emergence ages in most Pliocene and early Pleistocene taxa fall within African ape ranges, additional data are needed to discern how similar their life histories were. Future studies that aim to predict hominin life history would benefit from analyses of aspects of dental development that have been suggested to correlate with primate life history variables (e.g., molar crown formation time, molar eruption age, tooth calcification timing, long-period line periodicity) in samples of wild primates with documented life histories. These approaches would also benefit greatly from statistical approaches that control for phylogenetic relationships, as well as the potential confounding effect of sexual dimorphism and variation in tooth and body size.

While it is tempting to compare aspects of dental development in order to speculate on the taxonomic identity of StW 151 or KB 5223, additional data are required from securely identified Pliocene and early Pleistocene hominins. Little is known about dental development and overall tooth morphology in early *Homo* relative to species of *Australopithecus* or *Paranthropus* due to limited samples and uncertainty over the taxonomic identification of particular elements [56, 57, 100]. Moreover, most reports of early *Homo* cuspal enamel thickness and crown formation time involve analyses of naturally fractured teeth, which may be of limited comparative value due to section obliquity [15, 101], or the necessity of quantifying development from non-standardized positions on the tooth crown. Although marked differences in certain developmental features have been documented between chimpanzees and extant humans [51], orangutans and *Homo erectus* [102], and great apes and australopiths [52], considerable variation within and among fossil and extant species suggests that taxonomic interpretations of dental development in early hominins should be made with caution, if at all, as is the case with overall measures of enamel thickness [103].

## Supporting Information

S1 File Contains supporting tables and figures. **Fig. A**. Linear cuspal enamel thickness values in four hominin taxa. **Fig. B**. Long-period line periodicity in two *A*. *anamensis* individuals. **Fig. C**. Developmental plate used to assess tooth calcification in *A*. *anamensis* (KNM-KP 31712). **Fig. D**. Developmental plate used to assess tooth calcification in *A*. *anamensis* (KNM-KP 34725). **Fig. E**. Developmental plate used to assess tooth calcification in *A*. *africanus* (Sts 2). **Fig. F**. Developmental plate used to assess tooth calcification in *A*. *africanus* (Sts 24). **Fig. G**. Developmental plate used to assess tooth calcification in *A*. *africanus* (MLD 11/30). **Fig. H**. Developmental plate used to assess tooth calcification in *P*. *robustus* (SK 62). **Fig. I**. Developmental plate used to assess tooth calcification in *P*. *robustus* (TM 1536). **Fig. J**. Developmental plate used to assess tooth calcification in *P*. *robustus* (DNH 44). **Fig. K**. Developmental plate used to assess tooth calcification in *P*. *robustus* (DNH 47). **Fig. L**. Developmental plate used to assess tooth calcification in *P*. *robustus* (DNH 84). **Fig. M**. Developmental plate used to assess tooth calcification in *P*. *robustus* (DNH 107). **Fig. N**. Developmental plate used to assess tooth calcification in *P*. *robustus* (DNH 108). **Fig. O**. Developmental plate used to assess tooth calcification in early *Homo* (DNH 35). **Fig. P**. Developmental plate used to assess tooth calcification in early *Homo* (DNH 83). **Fig. Q**. Developmental plate used to assess tooth calcification in StW 151. **Fig. R**. Developmental plate used to assess tooth calcification in KB 5223. **Fig. S**. Ages at death predicted from modern human calcification standards compared to known- or histologically-derived ages. **Fig. T**. Recently erupted lower right first molar of DNH 107, a 4.8 year-old *P*. *robustus* individual from Drimolen. **Table A**. Long-period line periodicity (in days) for individuals in this study. **Table B**. Long-period line periodicity (in days) for fossil hominins, extant humans, and chimpanzees. **Table C**. Results of Mann-Whitney U test for comparisons of long-period line periodicity in *P*. *robustus* and *A*. *africanus* with fossil hominin taxa, extant humans, African apes, and previously published values. **Table D**. Cuspal enamel thickness values (in microns) for fossil hominins in this study. **Table E**. Age at death calculations for individual hominin specimens. **Table F**. Comparison of age at death estimates for two complete *P*. *robustus* mandibular dentitions. **Table G**. Histologically-determined initiation ages in Pliocene and early Pleistocene hominins.
(DOC)
