# Supplementary material for: Dental Ontogeny in Pliocene and Early Pleistocene Hominins
Source: PLoS One. 2015 Feb 18;10(2):e0118118. doi: 10.1371/journal.pone.0118118 (PMC4334485; doi:10.1371/journal.pone.0118118)
Supplement: S1 File — Fig. A. Linear cuspal enamel thickness values in four hominin taxa. Fig. B. Long-period line periodicity in two A. anamensis individuals. Fig. C. Developmental plate used to assess tooth calcification in A. anamensis (KNM-KP 31712). Fig. D. Developmental plate used to assess tooth calcification in A. anamensis (KNM-KP 34725). Fig. E. Developmental plate used to assess tooth calcification in A. africanus (Sts 2). Fig. F. Developmental plate used to assess tooth calcification in A. africanus (Sts 24). Fig. G. Developmental plate used to assess tooth calcification in A. africanus (MLD 11/30). Fig. H. Developmental plate used to assess tooth calcification in P. robustus (SK 62). Fig. I. Developmental plate used to assess tooth calcification in P. robustus (TM 1536). Fig. J. Developmental plate used to assess tooth calcification in P. robustus (DNH 44). Fig. K. Developmental plate used to assess tooth calcification in P. robustus (DNH 47). Fig. L. Developmental plate used to assess tooth calcification in P. robustus (DNH 84). Fig. M. Developmental plate used to assess tooth calcification in P. robustus (DNH 107). Fig. N. Developmental plate used to assess tooth calcification in P. robustus (DNH 108). Fig. O. Developmental plate used to assess tooth calcification in early Homo (DNH 35). Fig. P. Developmental plate used to assess tooth calcification in early Homo (DNH 83). Fig. Q. Developmental plate used to assess tooth calcification in StW 151. Fig. R. Developmental plate used to assess tooth calcification in KB 5223. Fig. S. Ages at death predicted from modern human calcification standards compared to known- or histologically-derived ages. Fig. T. Recently erupted lower right first molar of DNH 107, a 4.8 year-old P. robustus individual from Drimolen. Table A. Long-period line periodicity (in days) for individuals in this study. Table B. Long-period line periodicity (in days) for fossil hominins, extant humans, and chimpanzees. Table C. Results of Mann-Whitney U test fo [file pone.0118118.s001.doc]

**Supporting Tables**

Table A. Long-period line periodicity (in days) for individuals in this study.

It was not possible to determine the periodicity for juvenile DNH 83 or for isolated teeth DNH 39 and DNH 62. The periodicity for EM 2368 was originally reported in ref. 55; this is a fragment of a tooth from Swartkrans curated by the Natural History Museum (London), for which a SK number was not assigned after recovery.

Table B. Long-period line periodicity (in days) for fossil hominins, extant humans, and chimpanzees.

Note alternative values for means and ranges reflect uncertainty in the determination of individual periodicity values (e.g., *A. anamensis* KNM-KP 34725 is either 5 or 6 days, yielding a mean of 8 or 8.5 days).

Table C. Results of Mann-Whitney U test for comparisons of long-period line periodicity in *P. robustus* and *A. africanus* with fossil hominin taxa, extant humans, African apes, and previously published values.

Tests were conducted for species represented by four or more individuals. Sample sizes are given in Table B. Published values for *P. robustus* and *A. africanus* include eight individuals for each species. Significant results are in bold.

Table D. Cuspal enamel thickness values (in microns) for fossil hominins in this study.

See Table 2 for individual tooth and cusp abbreviations.

Table E. Age at death calculations for individual hominin specimens (part 1).

See Table 2 for individual tooth and cusp abbreviations. Cuspal time calculated by division of the cuspal enamel thickness by the species-specific or individually-measured daily secretion rate (DSR - given in first column). LP lines = long-period lines: Retzius lines or perikymata. CFT = crown formation time, determined at the sum of cuspal and lateral formation times. See methods for details about initiation ages and age at death calculations.

Table E. Age at death calculations for individual hominin specimens (continued).

Table F. Comparison of age at death estimates for two complete *P. robustus* mandibular dentitions.

Chimpanzee standards from ref. 79, human standards from ref. 71.

Table G. Histologically-determined initiation ages in Pliocene and early Pleistocene hominins.

See Table 2 for individual tooth and cusp abbreviations.

Figure A. Linear cuspal enamel thickness values in four hominin taxa.


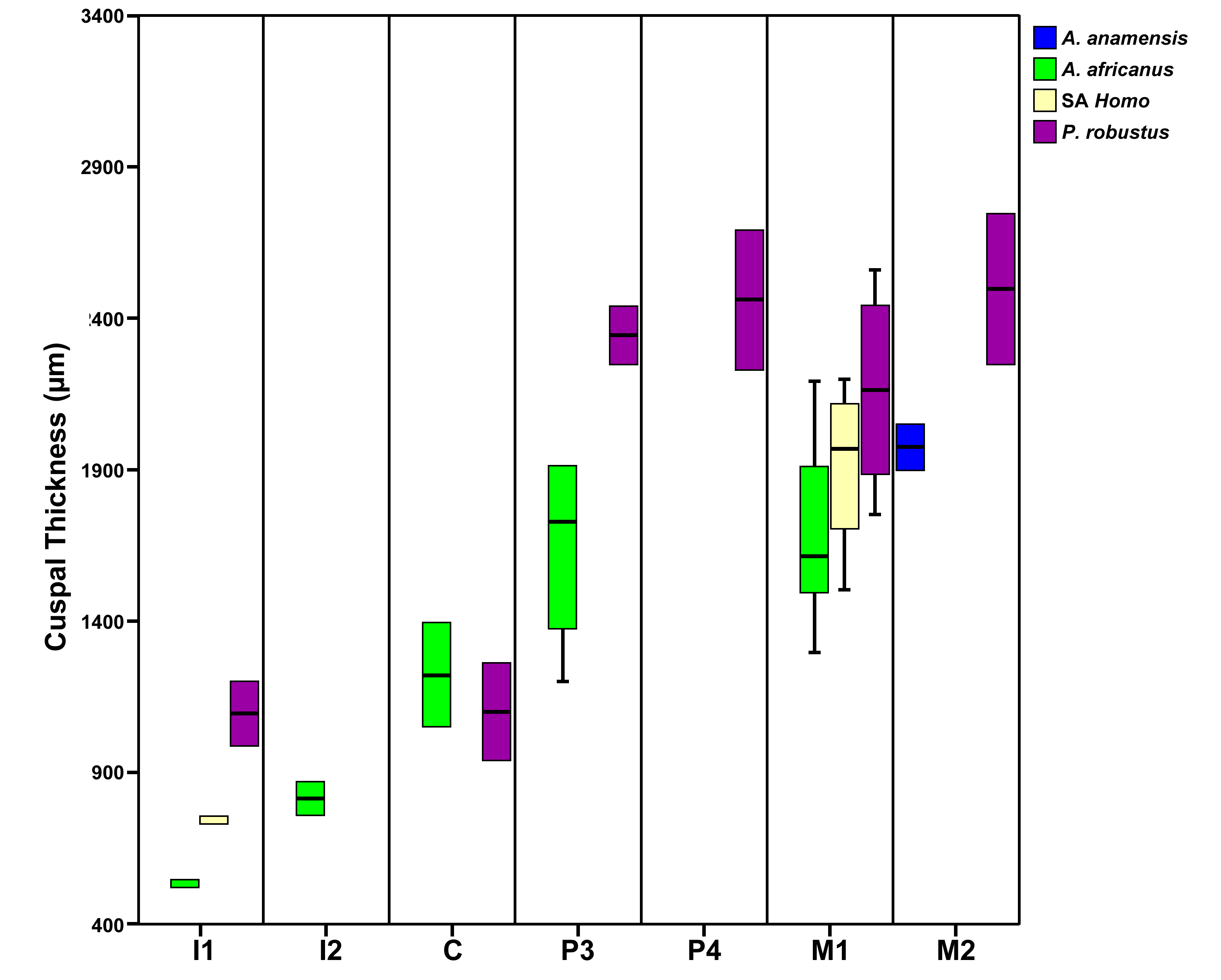


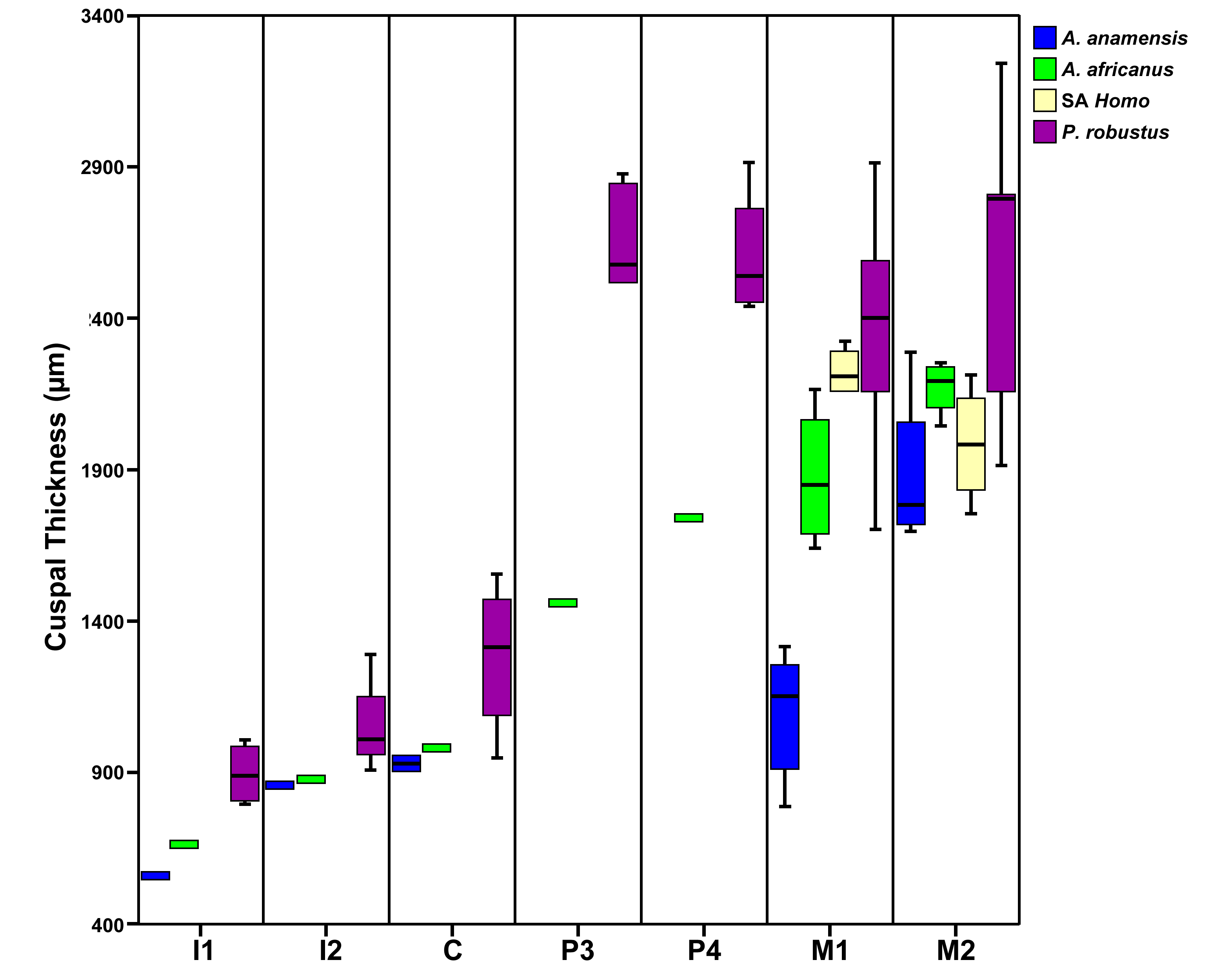


Values for premolars include both buccal and lingual cusps, values for molars include the four primary mesial and distal cusps. Individual values for specific cusps are given in Table D.

Figure B. Long-period line periodicity in two *A. anamensis* individuals.


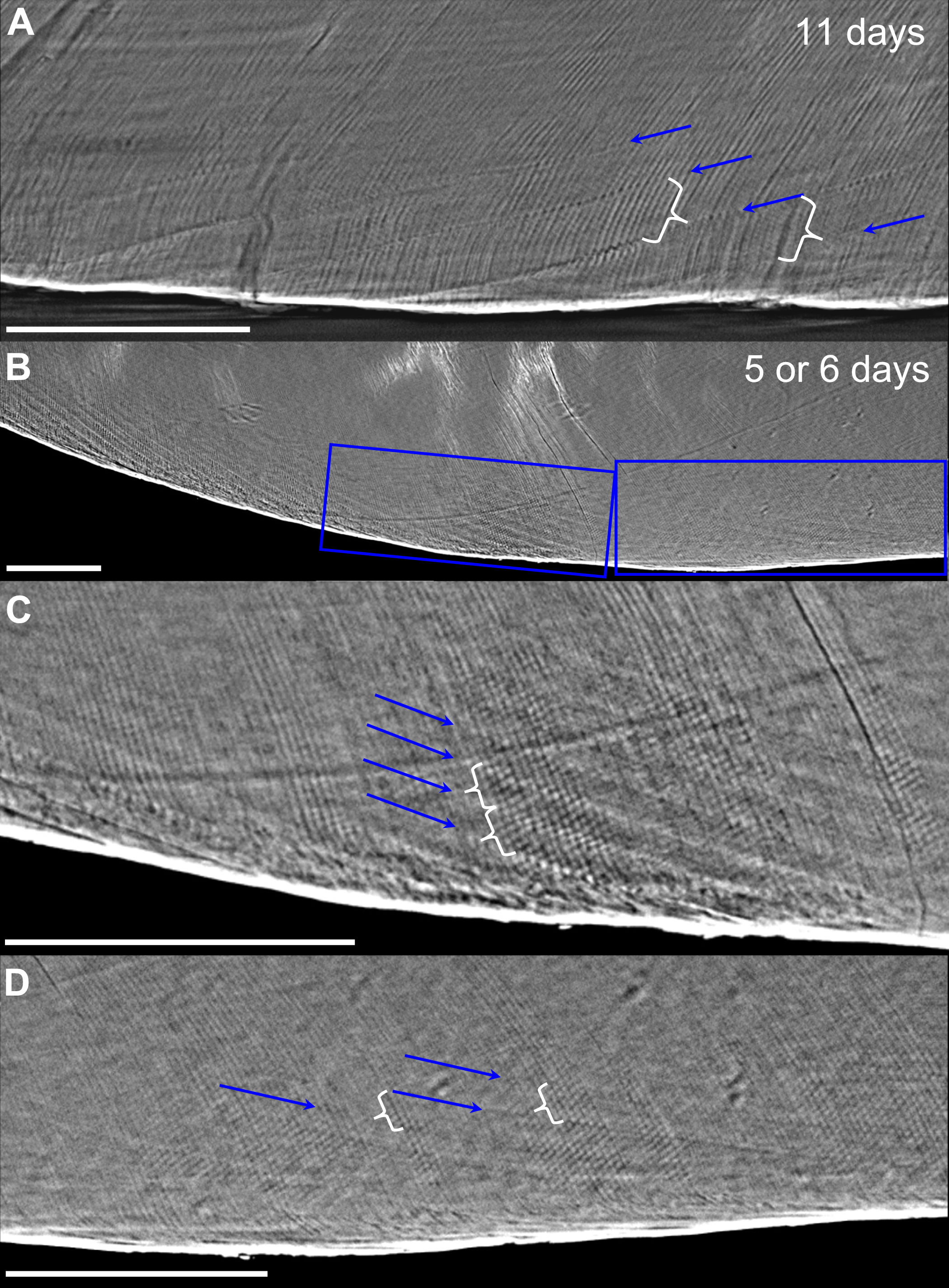


Long period lines are indicated with blue arrows, and white brackets indicate regions where a complete series of daily lines (paired light and dark bands) may be counted, yielding the long-period line periodicity in days. The scale bar in each image is equal to 0.2 mm. A. KNM-KP 31712, 11 day periodicity. B. KNM-KP 34725, 5 or 6 day periodicity. The blue box on the left shows the region enlarged in C, and the blue box on the right shows the region enlarged in D. It is not possible to conclude whether the correct value is 5 or 6 days.

Figure C. Developmental plate used to assess tooth calcification in *A. anamensis* (KNM-KP 31712).


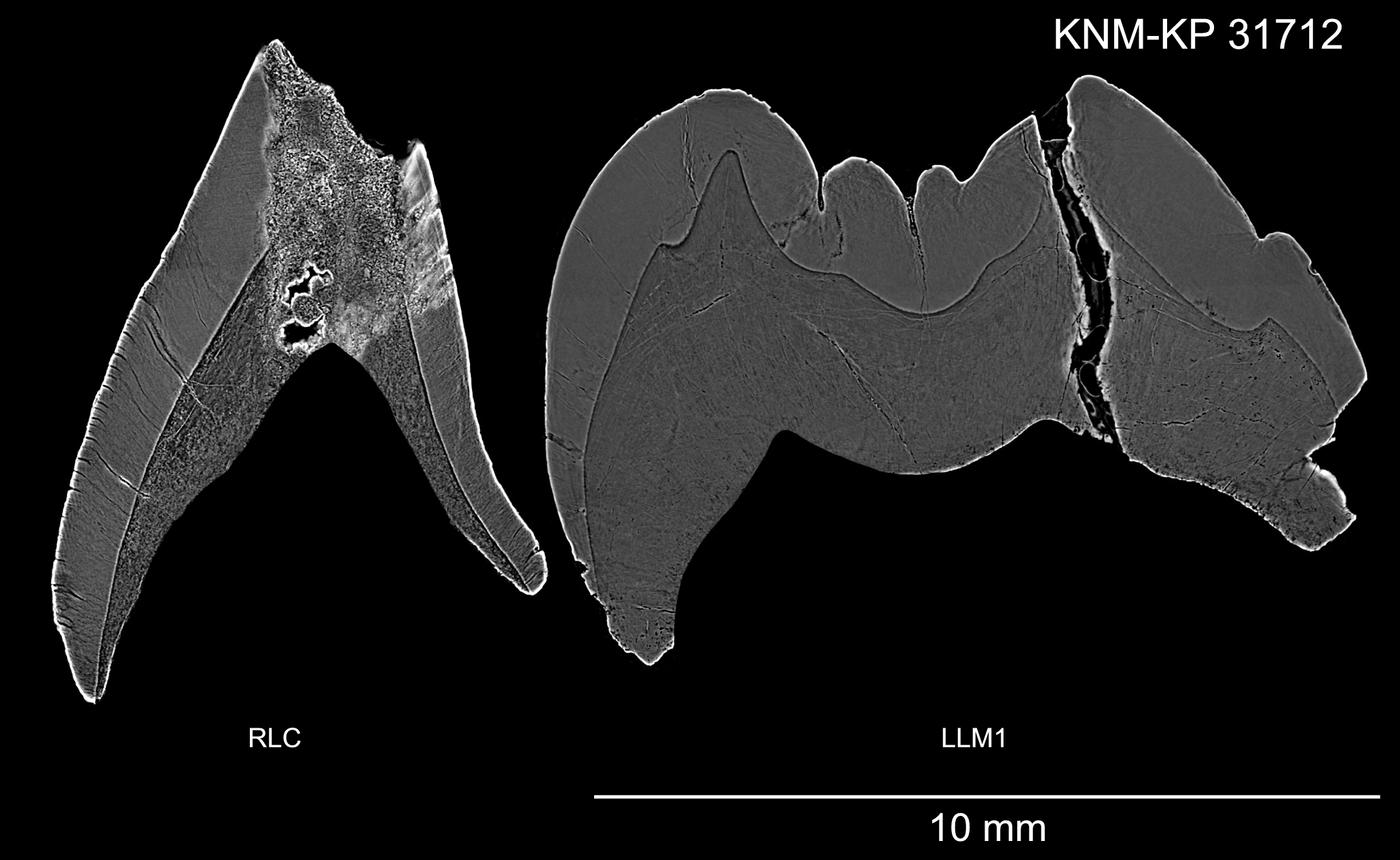


Figure D. Developmental plate used to assess tooth calcification in *A. anamensis* (KNM-KP 34725).


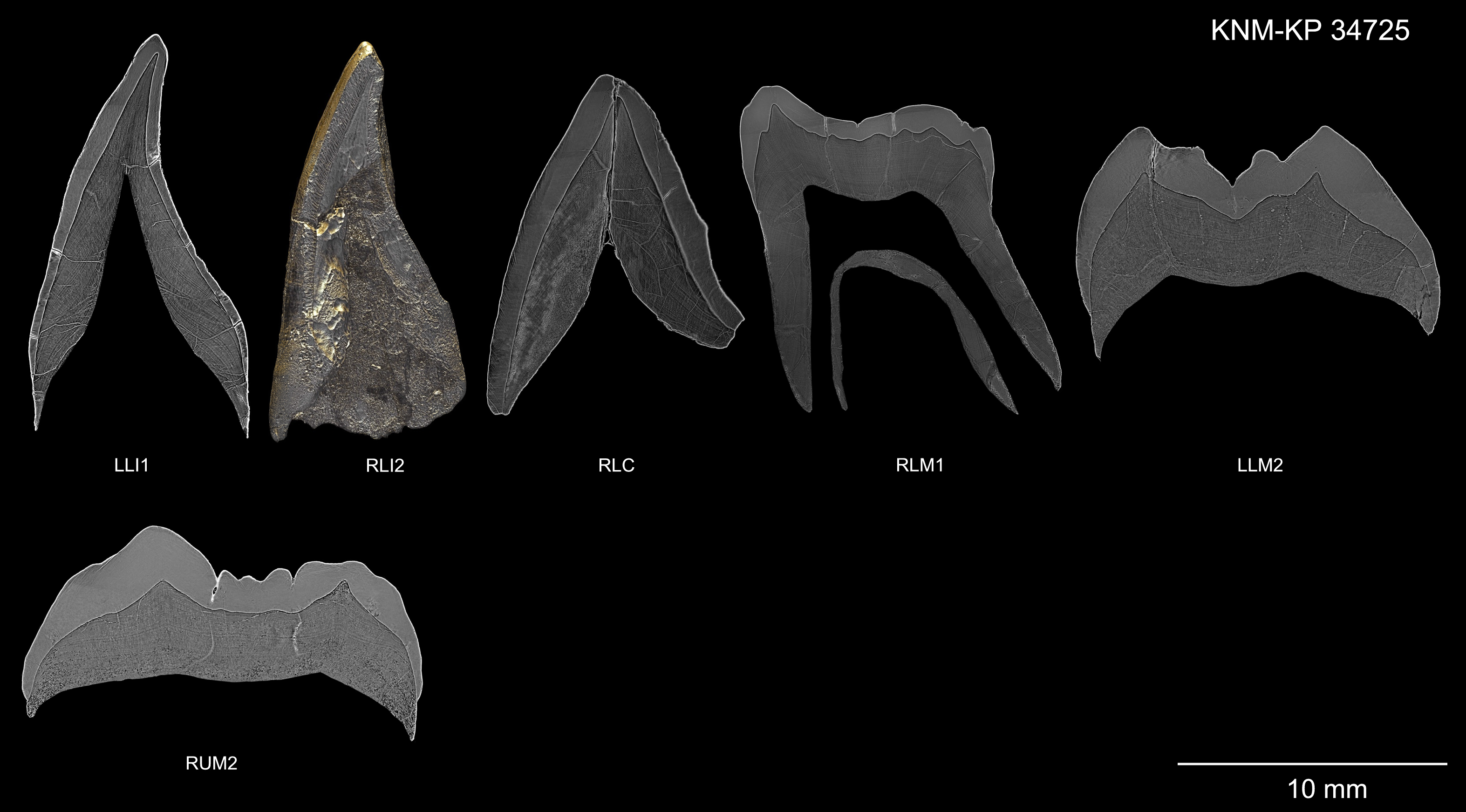


Note the RLI2 is a 3D model as the traditional 2D section plane has a fracture running down the tooth crown. The RLM1 was cut in a mesio-distal plane to show that the root apices are still open.

Figure E. Developmental plate used to assess tooth calcification in *A. africanus* (Sts 2).


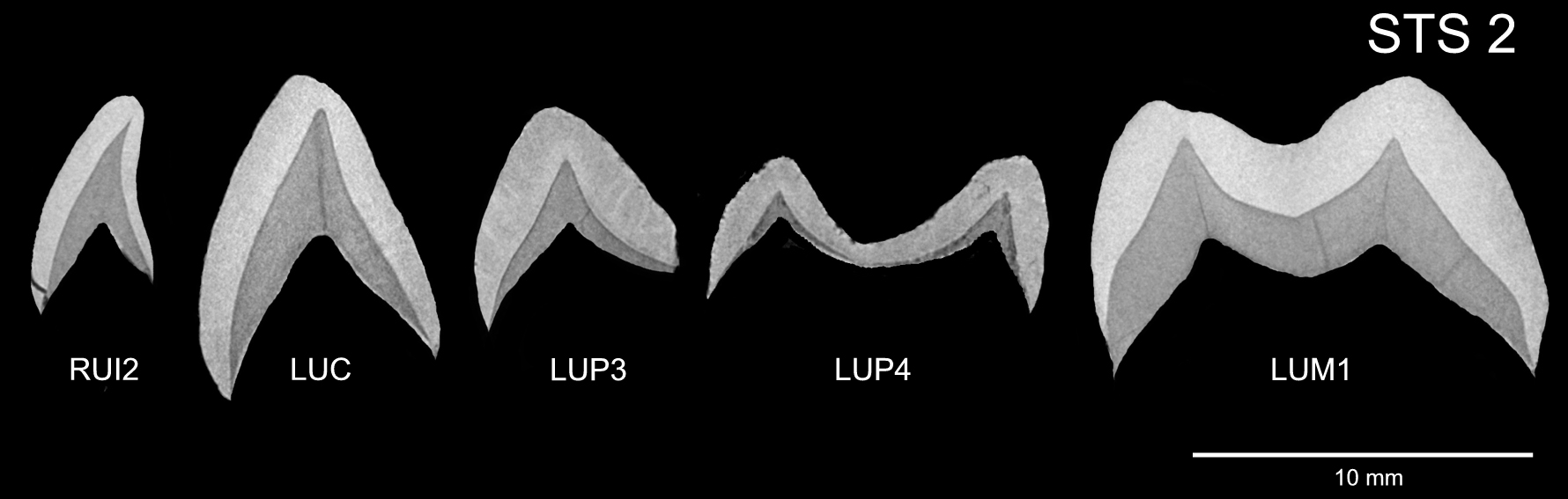


Figure F. Developmental plate used to assess tooth calcification in *A. africanus* (Sts 24).


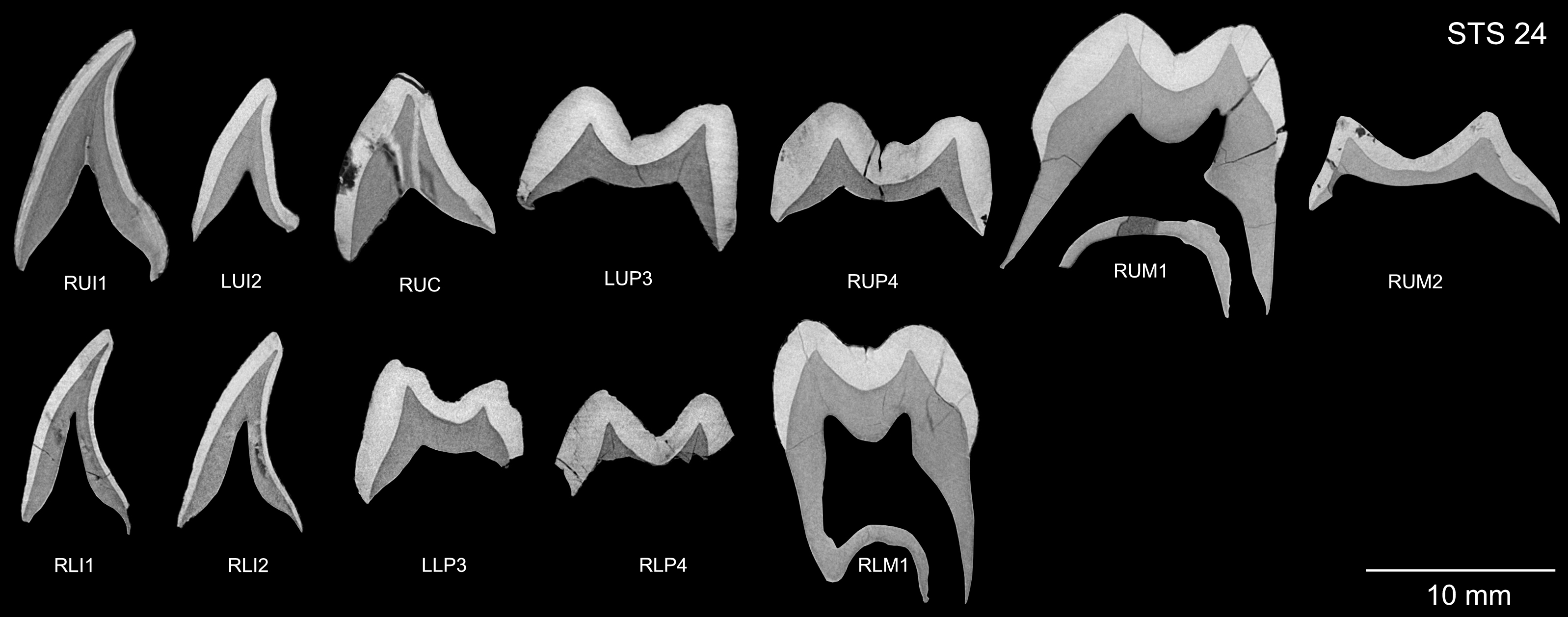


The RLP4 was not assessed due to the lack of preserved cervices.

Figure G. Developmental plate used to assess tooth calcification in *A. africanus* (MLD 11/30).


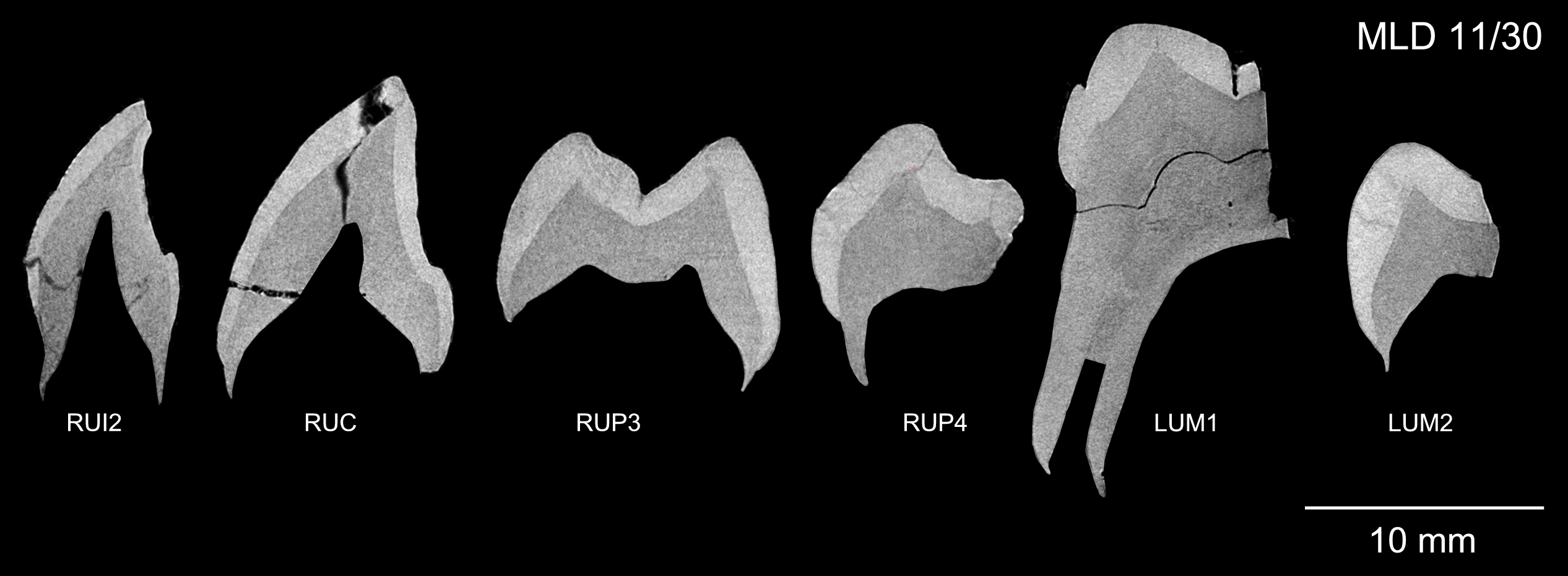


Figure H. Developmental plate used to assess tooth calcification in *P. robustus* (SK 62).


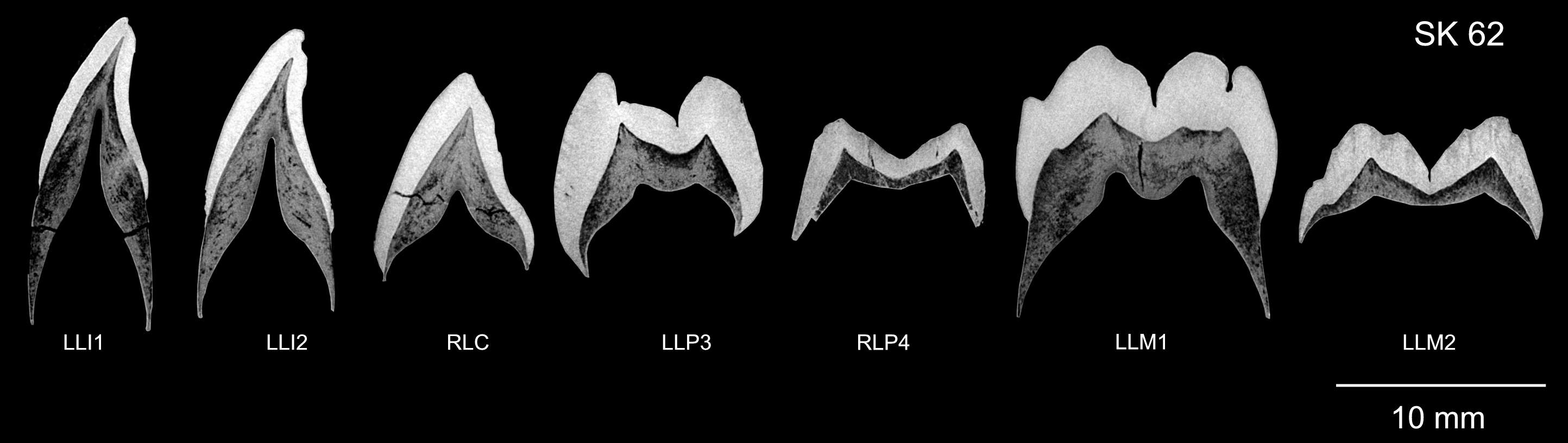


Figure I. Developmental plate used to assess tooth calcification in *P. robustus* (TM 1536).


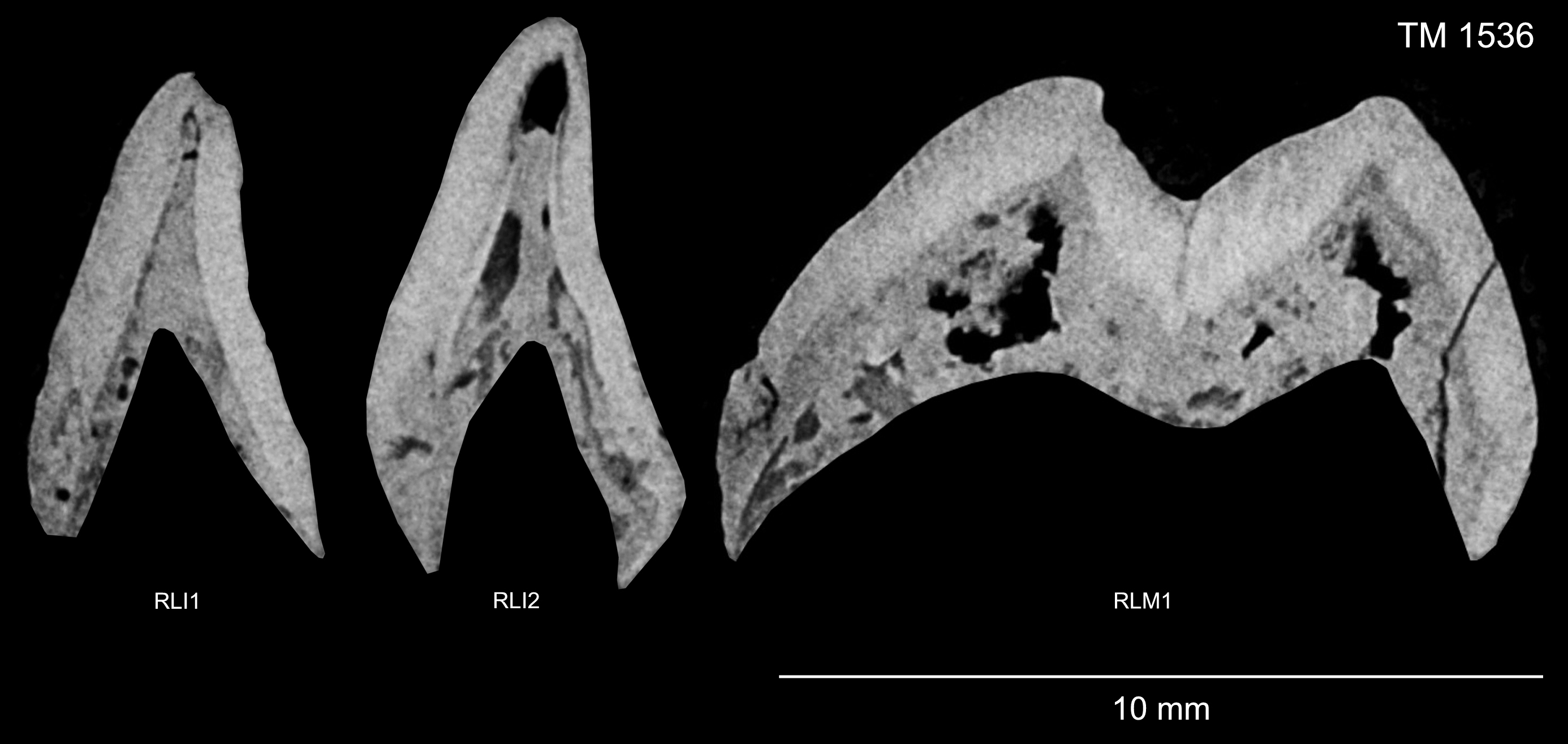


Figure J. Developmental plate used to assess tooth calcification in *P. robustus* (DNH 44).


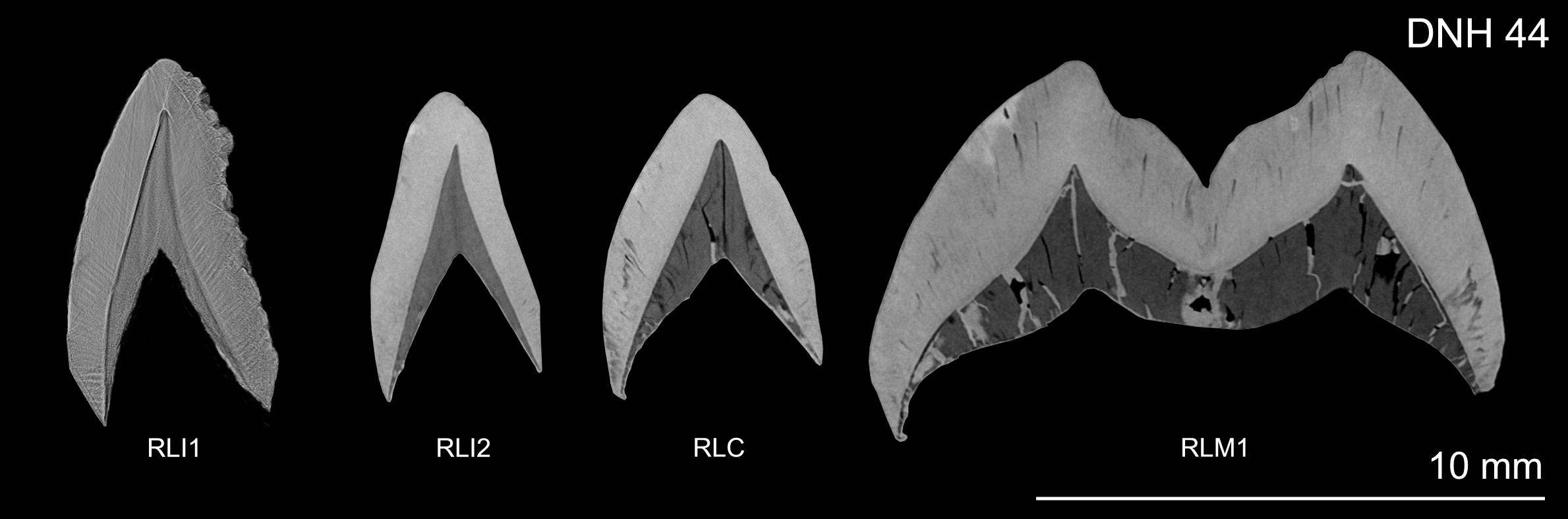


Figure K. Developmental plate used to assess tooth calcification in *P. robustus* (DNH 47).


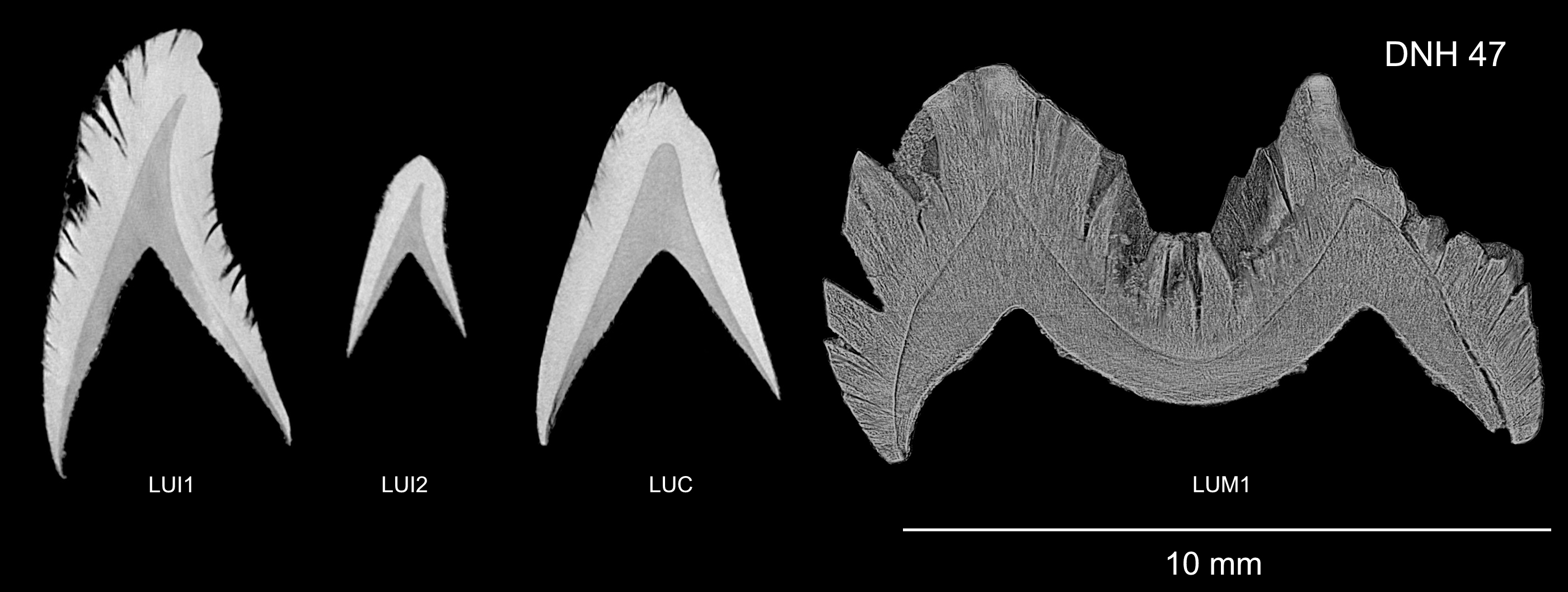


Figure L. Developmental plate used to assess tooth calcification in *P. robustus* (DNH 84).


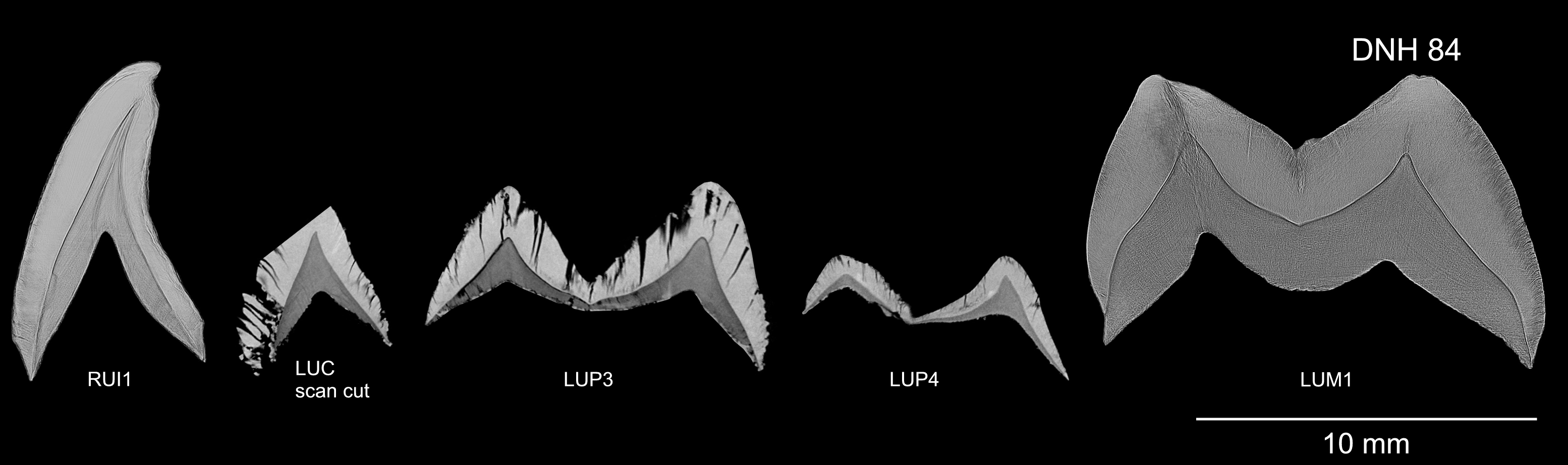


Figure M. Developmental plate used to assess tooth calcification in *P. robustus* (DNH 107).


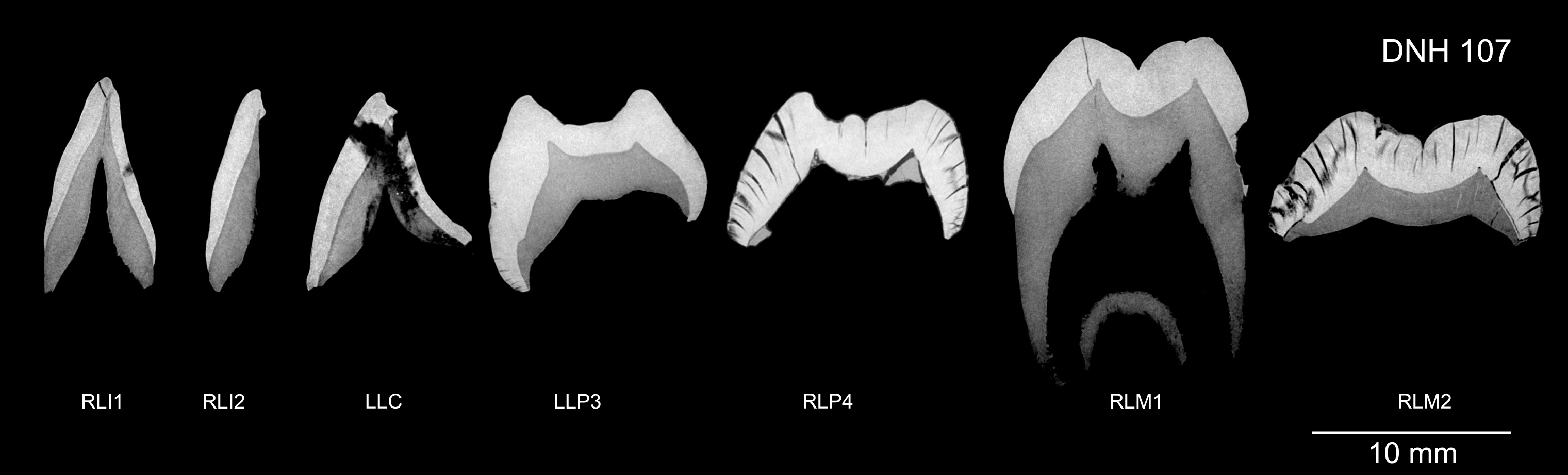


Figure N. Developmental plate used to assess tooth calcification in *P. robustus* (DNH 108).


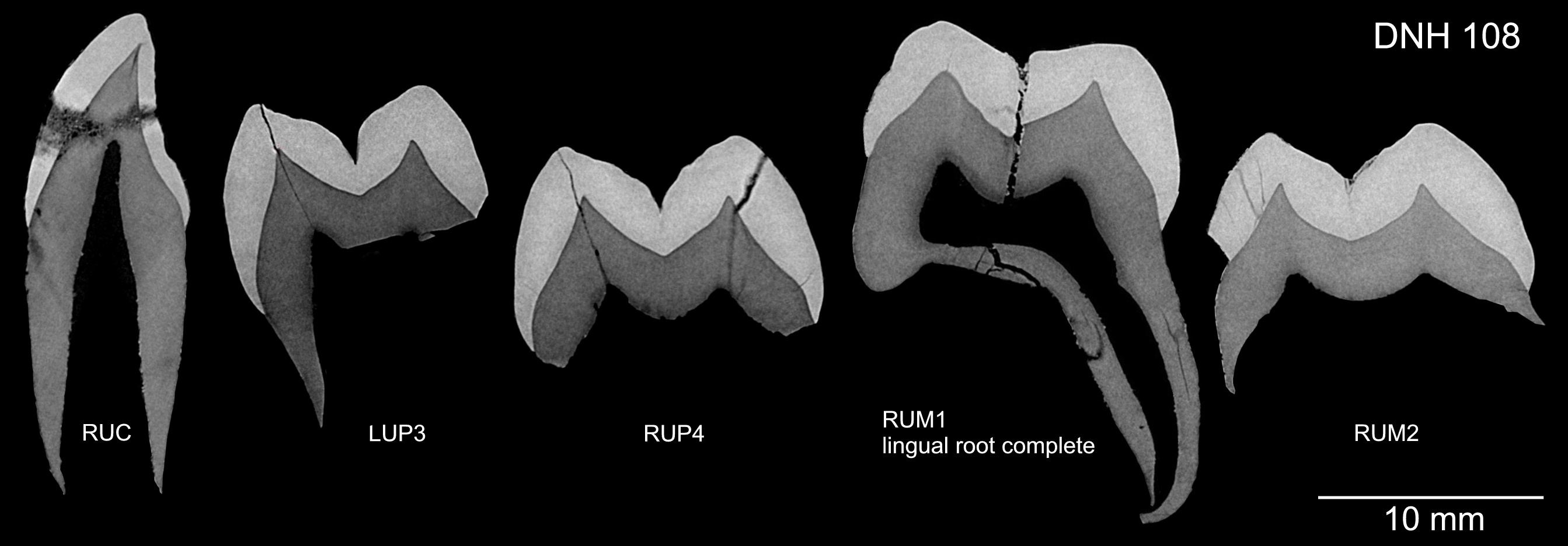


Figure O. Developmental plate used to assess tooth calcification in early *Homo* (DNH 35).


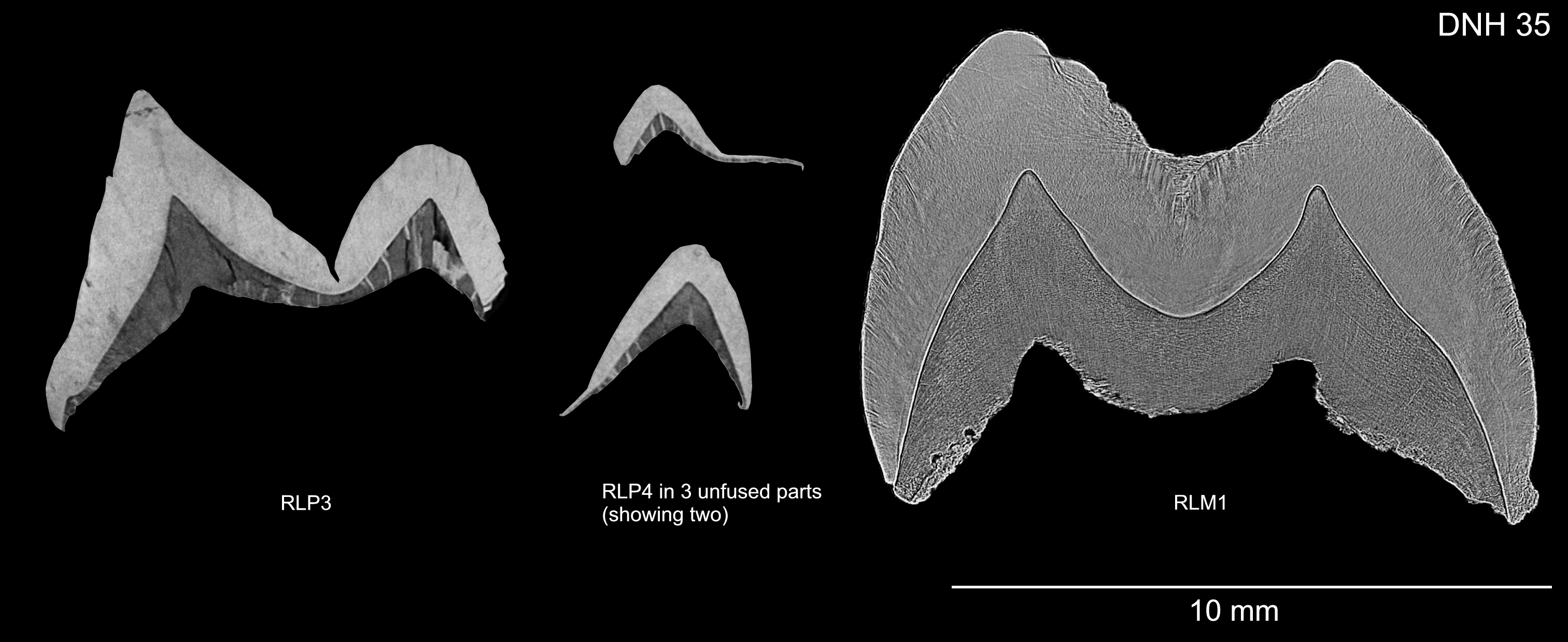


Figure P. Developmental plate used to assess tooth calcification in early *Homo* (DNH 83).


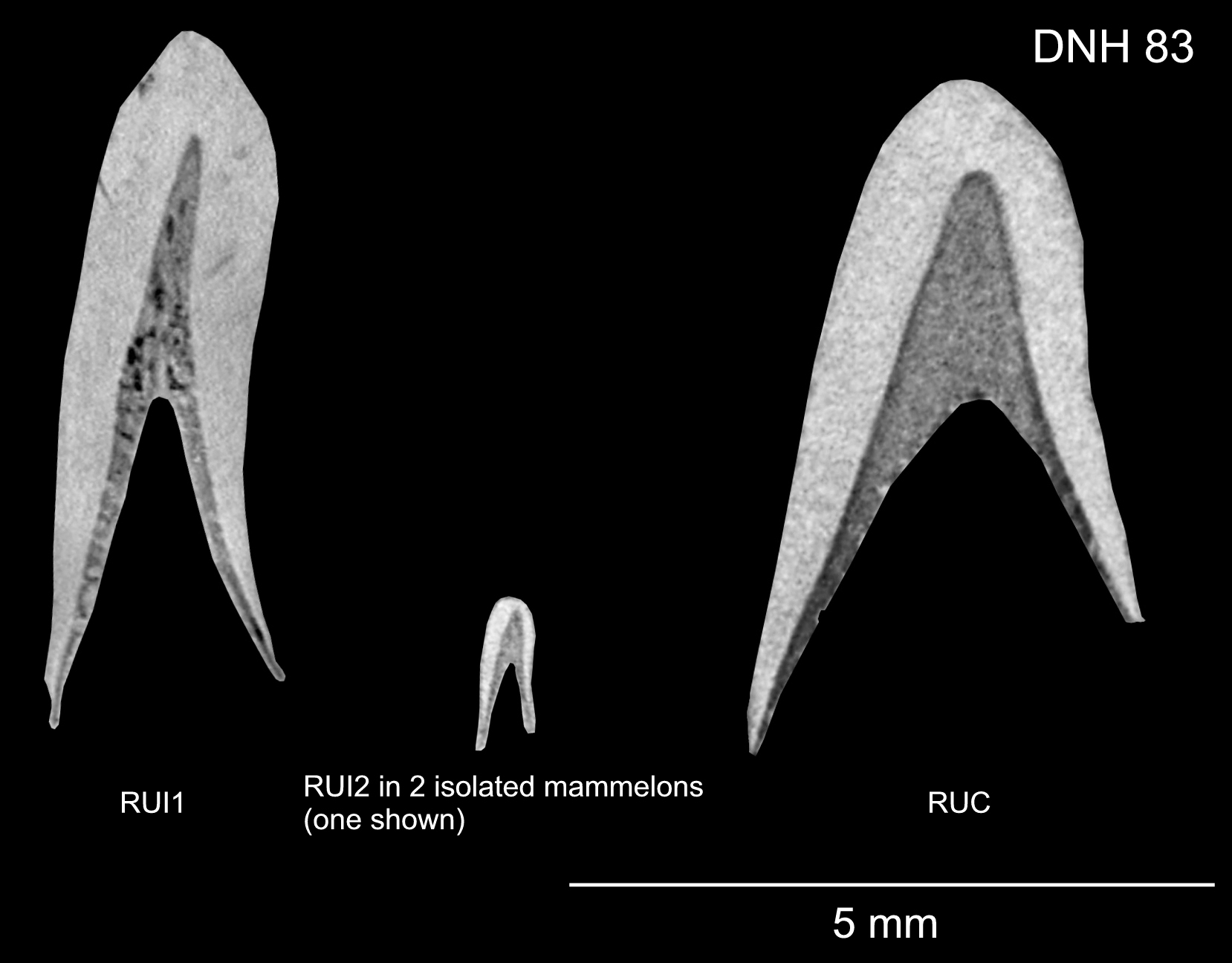


Figure Q. Developmental plate used to assess tooth calcification in StW 151.


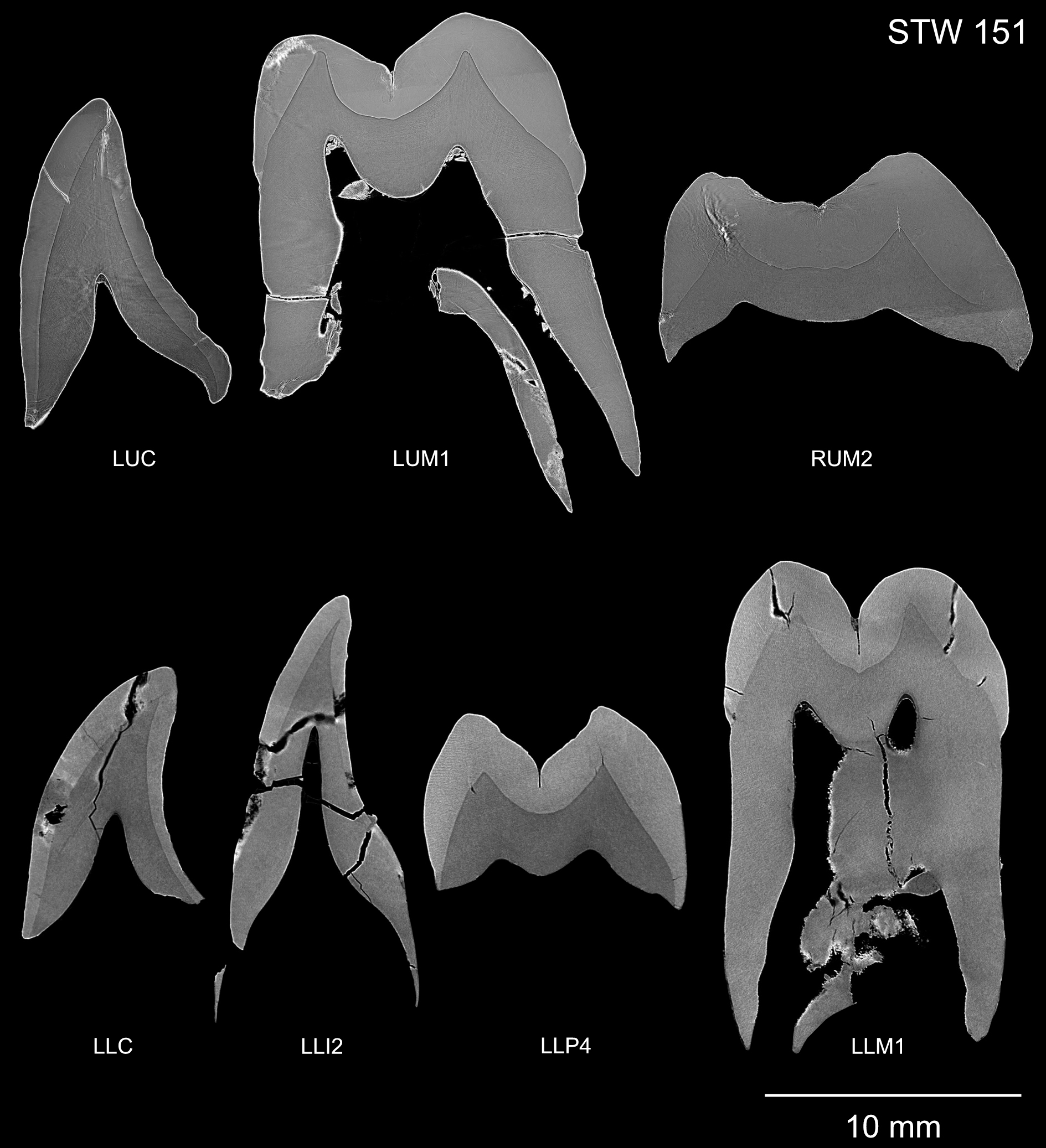


Figure R. Developmental plate used to assess tooth calcification in KB 5223.


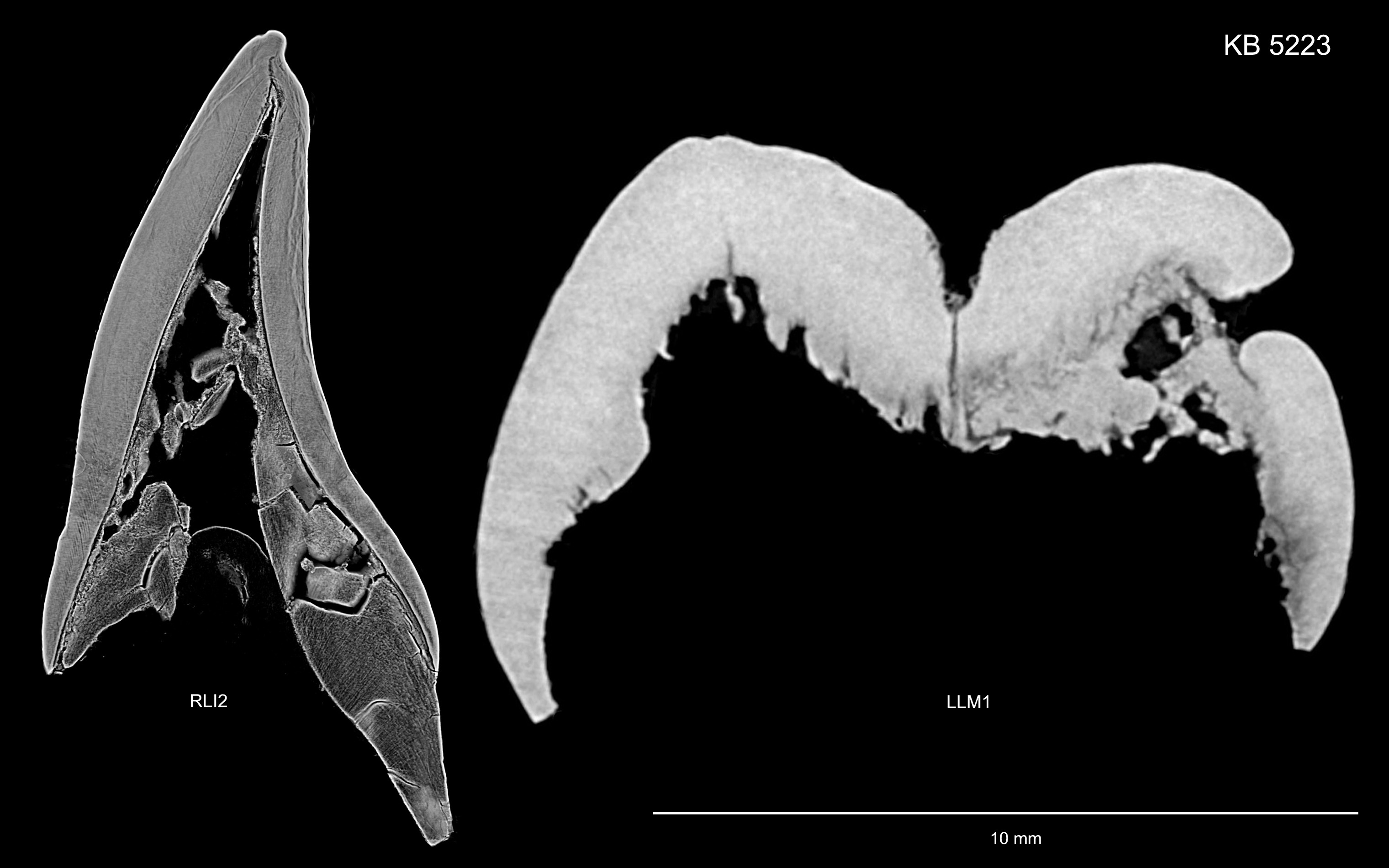


The LLM1 was deemed too modified to assess the calcification stage, and is shown for comparison only. While the mesial cusps were too altered for enamel thickness assessment, the distolingual cusp appeared to preserve the entire thickness of enamel, and was thus employed for age at death assessment.

Figure S. Ages at death predicted from modern human calcification standards compared to known- or histologically-derived ages.


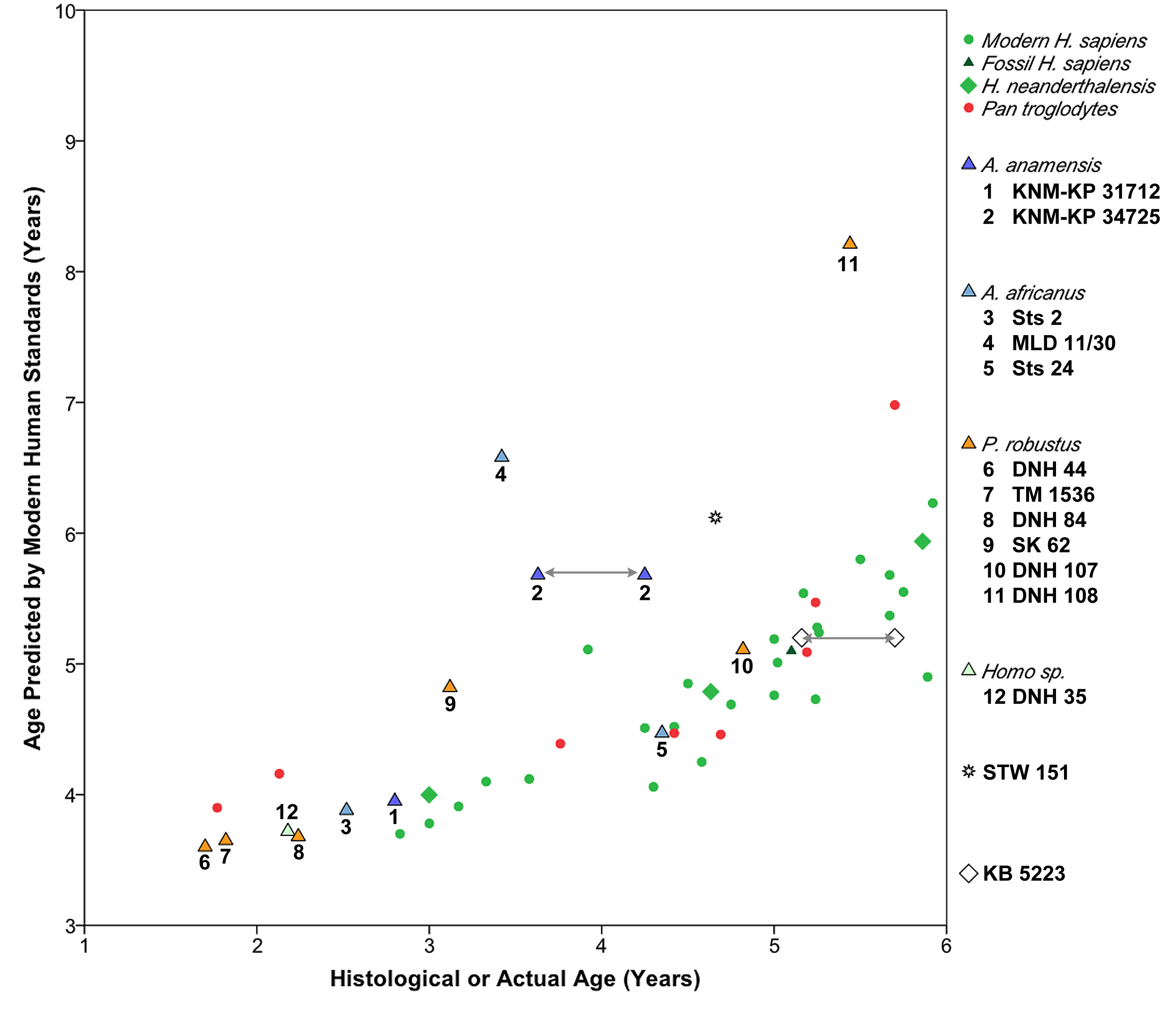


Two values are presented for *A. anamensis* KNM-KP 34725 due to uncertainty in the periodicity value. Data on extant human children derive from panoramic X-rays of known-age European and North African children, representing an expanded sample originally detailed in ref. 54. Fossil *Homo sapiens* and *Homo neanderthalensis* samples are from ref. 54; *Pan troglodytes* are known-age wild western chimpanzees [72].

Figure T. Recently erupted lower right first molar of DNH 107, a 4.8 year-old *P. robustus* individual from Drimolen.


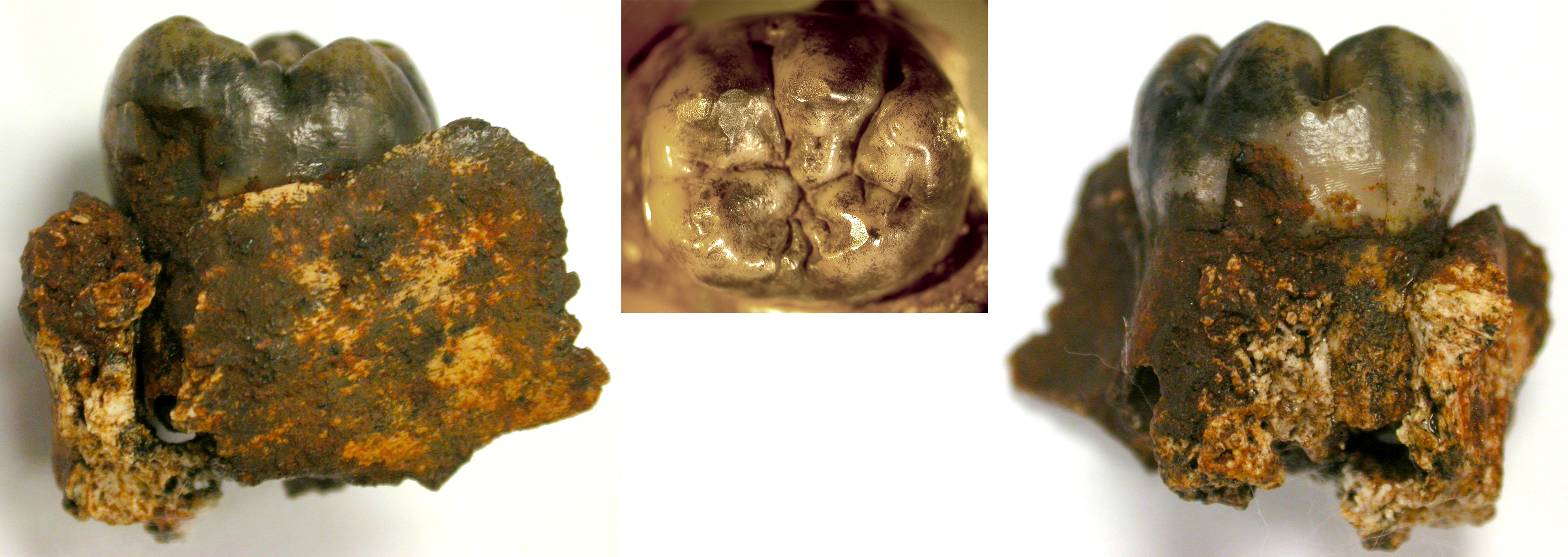


Left: buccal view; center: occlusal view; right: lingual view. Images are shown at the same scale.
